# Diverse Forms of *RPS9* Splicing Are Part of an Evolving Autoregulatory Circuit

**DOI:** 10.1371/journal.pgen.1002620

**Published:** 2012-03-29

**Authors:** Alex M. Plocik, Christine Guthrie

**Affiliations:** Department of Biochemistry and Biophysics, University of California San Francisco, San Francisco, California, United States of America; Princeton University, United States of America

## Abstract

Ribosomal proteins are essential to life. While the functions of ribosomal protein-encoding genes (RPGs) are highly conserved, the evolution of their regulatory mechanisms is remarkably dynamic. In *Saccharomyces cerevisiae*, RPGs are unusual in that they are commonly present as two highly similar gene copies and in that they are over-represented among intron-containing genes. To investigate the role of introns in the regulation of RPG expression, we constructed 16 *S. cerevisiae* strains with precise deletions of RPG introns. We found that several yeast introns function to repress rather than to increase steady-state mRNA levels. Among these, the *RPS9A* and *RPS9B* introns were required for cross-regulation of the two paralogous gene copies, which is consistent with the duplication of an autoregulatory circuit. To test for similar intron function in animals, we performed an experimental test and comparative analyses for autoregulation among distantly related animal *RPS9* orthologs. Overexpression of an exogenous *RpS9* copy in *Drosophila melanogaster* S2 cells induced alternative splicing and degradation of the endogenous copy by nonsense-mediated decay (NMD). Also, analysis of expressed sequence tag data from distantly related animals, including *Homo sapiens* and *Ciona intestinalis*, revealed diverse alternatively-spliced *RPS9* isoforms predicted to elicit NMD. We propose that multiple forms of splicing regulation among *RPS9* orthologs from various eukaryotes operate analogously to translational repression of the alpha operon by S4, the distant prokaryotic ortholog. Thus, *RPS9* orthologs appear to have independently evolved variations on a fundamental autoregulatory circuit.

## Author Summary

Eukaryotic genes are littered with non-coding intervening sequences, or introns, that must be precisely excised from a messenger RNA before it can be properly translated into protein. Despite their ubiquity, the evolution and function of introns remain poorly understood. Consequently, we cannot accurately predict the functions of individual introns in any organism. In this manuscript, we used a combination of comparative genomics and experimental tests to identify functional introns. First, we looked for signatures of selection to identify important introns in the model yeast *Saccharomyces cerevisiae*, which focused our attention on the introns of ribosomal protein genes. We then genetically deleted these introns to assess their function. Unlike mammalian introns, we found that yeast introns were not required for high levels of gene expression. Instead, particular introns (we focus on those within genes encoding ribosomal protein S9) were required to fine-tune gene expression through autoregulation. Surprisingly, animal orthologs of these genes also use introns to autoregulate through multiple forms of alternative splicing. We speculate that the introns of ribosomal protein genes, in particular, readily evolve means for autoregulation to meet the demanding requirements of ribosomal protein genes to maintain tight control of gene expression.

## Introduction

The evolution and function of spliceosomal introns are among the largest unsolved mysteries of eukaryotic genomes. Pronounced differences in intron evolution between lineages and between introns within the same lineage provide insight into 1) the selective and mutational forces governing intron evolution and 2) the potential roles of introns in gene function. Here we study the case of ribosomal protein genes (RPGs) in the model yeast *Saccharomyces cerevisiae*. RPGs are highly over-represented among intron-containing genes (69% of RPGs contain introns compared to ∼5% of non-RPGs), which has been suggested to reflect ongoing selection for introns that provide one or more functions in gene expression [1], [2]. However, two major facets of this hypothesis — the action of selection (intron evolution), and the source of this selection (intron function) — remain unknown. First, biased intron loss has not been specifically tested within hemiascomycetous yeasts (*S. cerevisiae* and relatives). And second, the effect of intron loss on RPG expression remains uncertain.

RPG expression is remarkable both in terms of synthesis rate and control [3]; thus, RPG introns may function to promote these aspects of gene expression. One proposal predicts that RPG introns function to promote high levels of expression. Consistent with this view, intron-containing genes, including RPGs, produce some of the highest transcript and protein abundances in *S. cerevisiae* [4]. However, the requirement for introns to enhance RPG expression has not been directly tested.

In addition to the above, two other proposals predict that RPG introns function by providing an opportunity for splicing regulation. One possibility is that introns provide rapid regulation in response to environmental stress, as suggested by splicing inhibition of RPG pre-mRNAs in response to amino acid starvation [5]. Another possibility is that introns provide an opportunity to fine-tune gene expression through autoregulation. For example, negative feedback control of *RPL30* and *RPS14B* expression is achieved through the binding of their respective protein products to RNA structures within their own unspliced transcripts, thereby regulating splicing [6], [7]. Interestingly, nearly all the ribosomal proteins of *Escherichia coli* are regulated by key ribosomal proteins in an analogous manner; for example, bacterial S4 directly binds its own mRNA to repress the translation of itself and three other RPGs [8], [9]. Given that the majority of *S. cerevisiae* RPGs contain introns, intron-dependent autoregulation may be more common than previously appreciated.

We report the first direct tests of both the action and the source of selection on RPG introns. First, we used comparative genomics to show that RPG introns have been preferentially retained following whole genome duplication (WGD), indicating ongoing selection for retention of RPG introns. Second, we generated *S. cerevisiae* strains harboring precise deletions of 16 RPG introns to distinguish between selective hypotheses. We found that RPG introns generally reduce gene expression, suggesting that RPG introns allow for splicing regulation rather than promoting high levels of expression. In particular, we identified intron-dependent cross-regulation between the *RPS9A* and *RPS9B* genes, which both encode ribosomal protein S9 (S9). Finally, overexpression of *RpS9* in *D. melanogaster* S2 cells, and analysis of available EST sequences, suggest that autoregulation of *RPS9* orthologs may involve different forms of splicing regulation between species, but also appears to be widespread across disparate lineages.

## Results

### Yeast ribosomal protein genes have resisted recent intron loss

Introns are over-represented in the RPGs of both *Candida albicans* and *S. cerevisiae* [1], [2]. While this shared over-representation may reflect selection pressure to maintain RPG introns prior to the divergence of these two species from a common ancestor, it may also reflect the action of selection in more recent history, since their divergence from a common ancestor. This distinction is important, since selection pressure to maintain RPG introns in more recent history is more likely to be relevant to the biology of *S. cerevisiae*. To determine if RPGs have resisted intron loss compared to other genes since the divergence of *C. albicans* and *S. cerevisiae* (∼200–800 million years ago [10]), we assessed the fates of *S. cerevisiae* introns in paralogs (a.k.a. gene pairs) that were duplicated ∼100 million years ago by whole-genome duplication (WGD) [11]. To determine the fates of introns after genome duplication, we took advantage of the well-annotated genome of *S. cerevisiae*, which has been exhaustively searched for introns [12], [13]. With these annotations, we identified 121 intron-containing genes among 554 WGD-derived gene pairs obtained from Yeast Gene Order Browser [14]. Assuming that intron loss has largely dominated intron evolution in hemiascomycetous yeast species [15], we inferred intron loss if one of the WGD-derived gene copies had fewer introns than the other. Using this criterion, we calculated the number of apparent intron losses in RPG pairs compared to all other gene pairs. Strikingly, this simple accounting revealed that 16 of 23 non-RPG pairs have a gene with fewer introns than its copy, whereas none of the 46 RPG pairs did. Nonetheless, this analysis ignores intron losses that occurred independently in both gene copies and assumes that intron gain did not occur.

**Figure 1:**
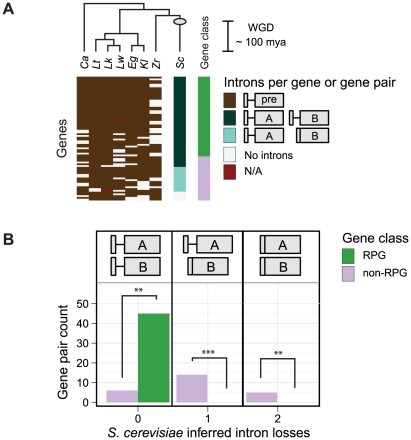
Biased intron loss in hemiascomycetous yeasts after the recent whole-genome duplication event. A) The dendogram (top) illustrates the assumed topology of the phylogenetic relationships used to infer intron-containing genes present in the pre-WGD ancestor prior to the WGD event (based on [63]; not to scale); the estimated time of the WGD event is indicated (gray circle and scale bar) [11]. A heatmap (bottom) illustrates the number of introns in *S. cerevisiae* gene pairs and their orthologs (rows) by species (columns). Genes containing an intron in pre-WGD species (brown tiles) were used to infer the intron-containing genes present in the pre-WGD ancestor (see Methods). Among the 95 *S. cerevisiae* gene pairs derived from an intron-containing gene in the pre-WGD ancestor, those with an intron in both gene copies (dark blue-green) were inferred to have no intron losses. *S. cerevisiae* gene pairs with an intron in only one of two gene copies (light blue-green) or no intron in either gene copy (white) were inferred to have had one or two intron losses, respectively. Missing genes (red) are indicated. RPGs (green) and other functional gene classes (purple) are indicated (right-most column). See Table S1 for intron predictions and annotations. *Ca = C. albicans*, *Lw = L. waltii*, *Lt = L. thermotolerans*, *Lk = L. kluyveri*, *Eg = E. gossypii*, *Kl = K. lactis*, *Zr = Z. rouxii*, *Sc = S. cerevisiae*. B) A histogram counts the number of inferred intron losses for each *S. cerevisiae* gene pair that descended from an intron-containing pre-WGD ortholog. Intron losses from RPGs (green) are compared to other functional gene classes (purple). Asterisks indicate statistical significance values p<0.01 (**), and 0.001 (***); exact binomial test.

To better assess whether WGD-derived RPG pairs have been biased for either intron gain or loss (including losses in both gene copies), we reconstructed the hypothetical intron distribution of the pre-WGD ancestor that existed prior to the WGD event. For each of the 554 *S. cerevisiae* duplicated gene pairs, we assigned the presence or absence of an intron in the hypothetical pre-WGD ancestral ortholog based on intron annotations and predictions from the genomes of the pre-WGD (so-called protoploid) species (*C. albicans*, *Lachancea waltii*, *L. thermotolerans*, *L. kluyveri*, *Eremothecium gossypii*, *Kluyveromyces lactis*, and *Zygosaccharomyces rouxii*) and the genomes of the post-WGD species (*Vanderwaltozyma polyspora*, *Naumovia castellii*, *C. glabrata*, and *S. bayanus*). A complete list of intron predictions and annotations can be found in Table S1. Our analysis revealed 73 intron-containing genes that were likely present in the pre-WGD ancestor from which the duplicated gene pairs in *S. cerevisiae* were descended (Figure 1A). Based on this hypothetical intron distribution of the pre-WGD ancestor, we inferred the number of *S. cerevisiae* WGD-derived gene pairs that have gained or lost an intron for each post-WGD gene pair (Figure 1B). From this improved analysis, we identified 5 *S. cerevisiae* non-RPG pairs that appear to have independently lost introns from both gene copies after gene duplication. This was in addition to 14 non-RPG pairs in which one of two introns were lost (Figure 1B, right and middle columns, respectively). Once again, we inferred no intron losses in *S. cerevisiae* RPG pairs (Figure 1B, left column). Thus, RPG introns appear to have been biased against loss in the lineage leading to *S. cerevisiae* during the last ∼100 million years.

Next, we asked whether intron gains contributed to the bias for introns in *S. cerevisiae* RPGs. For a given *S. cerevisiae* gene, we inferred that an intron was gained if introns were absent in both the pre-WGD ancestor and the majority of post-WGD orthologous gene pairs. Using this criterion, we did not infer intron gains in any of the *S. cerevisiae* RPGs. On the other hand, two introns in non-RPGs (i.e. *USV1* and *BMH2*) have possibly been gained in the *S. cerevisiae* lineage (Table S1); however, since both of these introns are located in the 5′ UTR and are not well annotated in other species, it is therefore difficult to be confident of this conclusion. Taken together, the bias for introns in *S. cerevisiae* RPG pairs appears to have been dominated not by intron gains in RPGs, but by intron losses in non-RPGs.

### Introns repress ribosomal protein gene expression

Having found a bias against RPG intron loss, we sought to determine if RPG introns have a function in gene expression. To mimic the effect of RPG intron loss, we created *S. cerevisiae* mutant intron deletion strains (henceforth denoted as Δi). Each Δi mutant was created with a precise deletion of a single RPG intron, such that only an intronless copy of the gene remained at the endogenous locus (See Methods).

**Figure 2:**
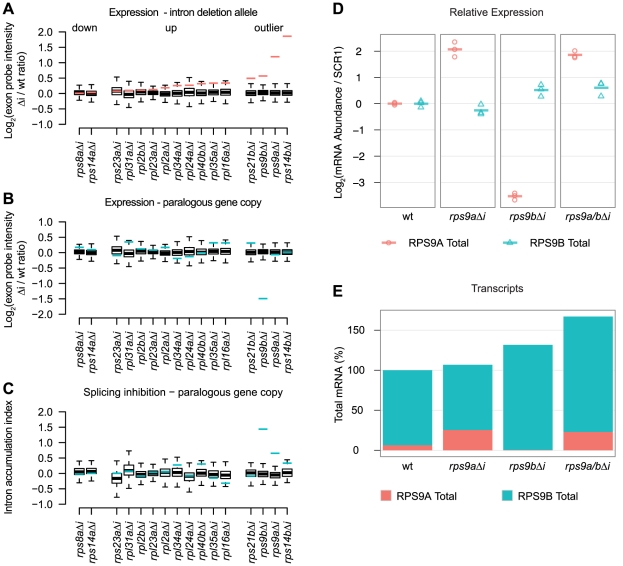
RPG intron deletions reveal gene-specific effects on steady-state mRNA levels. A–C) Microarray expression data for 16 RPG Δi mutants compared to a common wild-type strain. In each panel, the change in expression due to intron deletion is shown for either the intronless gene (red lines) or its paralogous gene copy (blue lines) compared to all other changes detected by microarray (boxplots). The effect of intron deletion is shown for each Δi mutant on A) the expression of the intronless gene copy, B) the expression of the paralogous gene copy, and C) the Intron Accumulation Index of the paralogous gene copy. Microarray data are expressed as the normalized log_2_ transformed probe intensity for exon features averaged from at least two replicate microarrays. Whiskers represent 1.5 times the interquartile range. D) RT-qPCR quantification of *RPS9A* (red circles) and *RPS9B* (blue triangles) expression changes for each Δi mutant relative to wild-type (columns). *RPS9A* and *RPS9B* values were divided by SCR1 values to obtain ratios controlled for variations in cDNA quantity. Log_2_ transformed ratios are plotted relative to wild-type (based on the mean of three biological replicates). Each of three biological replicates is shown as a point and the mean as a dash. E) The effect of intron deletion on the total number of transcripts encoding S9. Stacked barplots illustrate the percent of *RPS9A* (red bars) and *RPS9B* (blue bars) transcripts calculated for each Δi mutant. For a wild-type strain (first column), the percent of *RPS9A* and *RPS9B* transcripts encoding S9 were estimated from published RNA-seq data [19]. Changes in *RPS9A* and *RPS9B* transcript numbers for each Δi mutant (columns) were calculated by multiplying wild-type percentages by relative expression changes determined by qPCR.

Because RPGs are among the most highly expressed genes in the genome, we tested the model that introns are required in *cis* for high levels of gene expression by assessing the expression profiles of 16 Δi mutants compared to a wild-type strain. We also considered the possibility that Δi mutations may affect other genes in *trans*, in particular, the WGD-derived gene copies of RPG pairs. To measure changes in expression of the gene from which an intron was deleted (in addition to 124 RPG and 911 non-RPG features) we used custom splicing-sensitive microarrays designed to detect pre-, mature, and total mRNA species (using intron, junction, and exon probes, respectively [16]). To assess the effect of Δi mutation on gene expression, we plotted the expression change for the intronless gene (Figure 2A, red lines) compared to all the other genes on the microarray (Figure 2A, boxplots). Thus, the most significant expression changes lie outside the whiskers of the boxplot and are, by definition, statistical outliers. Intron deletion mutations, as assessed by microarray, typically had only modest effects on gene expression (Figure 2A, compare red lines to boxplots). Nonetheless, these effects were biased toward increased expression of the intronless gene (14 out of 16), rather than decreased expression (Figure 2A “up” and “down,” respectively). Moreover, the four most substantial expression changes increased the expression of the intronless gene (Figure 2A “outlier”). These data suggest that yeast introns are generally not required for the high expression levels of RPGs. Further, only a few genes showed substantial increases in expression, which suggests that splicing may be more inefficient for these genes than most other RPGs.

We also sought to determine if any of the deleted introns were required for splicing regulation. As controls, we deleted the introns of *RPS14A* and *RPS14B*, as it has been known for some time that S14 binds to the *RPS14B* intron (but not the *RPS14A* intron) to inhibit splicing and to cause rapid degradation [7], [17]. As expected, deletion of the *RPS14B* intron led to a substantial increase in its expression compared to the other genes on the microarray (Figure 2A “outlier”), whereas deletion of the *RPS14A* intron had little effect on expression (Figure 2A “down”). Thus, our microarrays have the sensitivity required to detect the derepression of *RPS14B* expression. An unexpected and novel finding is the substantial effect that Δi mutations have on the expression of the two gene copies encoding ribosomal protein S9 (hereafter referred to as S9). Our microarray experiments revealed that *RPS9A* and *RPS9B* Δi mutations increased the expression of the intronless genes (Figure 2A “outlier”) and also decreased the expression of the wild-type gene copies (Figure 2B). We hypothesized that the decreased expression of the wild-type *RPS9A* and *RPS9B* genes was caused by decreased splicing efficiency due to negative feedback. Therefore, we tested whether Δi mutations caused an increase in the ratio of pre-mRNA to total mRNA of the wild-type gene copies by calculating the Intron Accumulation Index of these genes, which is a measure of inefficient splicing [18]. Of all the mutants tested by microarray, only *RPS9A* and *RPS9B* showed substantial increases in the Intron Accumulation Index compared to the other intron containing genes on the array (Figure 2C, compare blue lines to boxplots). Taken together, these data suggest that the *RPS9A* and *RPS9B* genes require introns to repress their own expression. Further, derepression of *RPS9A* resulted in increased repression of *RPS9B* through splicing inhibition (and vice versa), suggesting that these genes cross-regulate.

Our custom microarray platform is precise; however, it lacks control probe sets needed for highly accurate quantification. As such, our microarrays “compress” fold-changes compared to equivalent determination by qPCR. To validate our most surprising observations, we assessed *RPS9A* and *RPS9B* expression by RT-qPCR. Importantly, we designed at least one qPCR primer to the 3′UTR in an effort to maximize specificity and to minimize artifacts caused by primer cross-hybridization to the other gene copy. As expected, qPCR measurements validated our microarray results for both *RPS9A* and *RPS9B* genes in the *rps9bΔi* and *rps9bΔi* mutants (Figure 2D, second and third columns). In the case of the *rps9aΔi* mutant, Δi mutation was associated with a substantial increase (>4-fold of wild-type) in *RPS9A* expression and a modest decrease (<2-fold of wild-type) in *RPS9B* expression (Figure 2D, second column). Conversely, in the *rps9bΔi* mutant, Δi mutation was associated with a modest increase (<2-fold of wild-type) in *RPS9B* expression and a substantial decrease (>8-fold of wild-type) in *RPS9A* expression (Figure 2D, third column).

Having validated the surprising effects of deleting the *RPS9A* and *RPS9B* introns, we hypothesized that the genes reciprocally cross-regulate through a shared negative feedback circuit. We made two strong predictions from this hypothesis: 1) deletion of both the *RPS9A* and *RPS9B* introns should eliminate cross-regulation, and therefore, derepress both gene copies and 2) the wild-type gene copy should compensate for a derepressed copy by an equal and opposite number of transcripts. First, to determine if repression of *RPS9A* expression in the *rps9bΔi* mutant required the *RPS9A* intron (and vice versa), we created a double *rps9a/bΔi* mutant and tested the effect on expression by RT-qPCR. As predicted, both *RPS9A* and *RPS9B* were derepressed in the *rps9a/bΔi* mutant (Figure 2D, fourth column). Second, we sought to determine if changes in the number of *RPS9A* transcripts were compensated by a nearly equal and opposite change in number of *RPS9B* transcripts. We first estimated the percent of transcripts encoding S9 contributed by the *RPS9A* and *RPS9B* genes (6% and 94%, respectively) from a published RNA-seq data set from a wild-type strain [19]. In order to calculate the number of transcripts in each Δi mutant, we then simply multiplied the percent of transcripts encoding S9 (as determined by RNA-seq) by the relative change in expression (as determined by qPCR) for each Δi mutant. As predicted for the *rps9aΔi* mutant, a substantial relative increase in *RPS9A* expression mutant was nearly equally compensated by a modest relative decrease in *RPS9B* expression, such that the total number of transcripts encoding S9 was nearly unchanged (Figure 2D and 2E, second column). In the *rps9bΔi* mutant, however, a modest relative increase in *RPS9B* expression mutant was only partially compensated at the expense of nearly all *RPS9A* transcripts (Figure 2D and 2E, second column). In this case, it appears that *RPS9A* defied our prediction and presumably because its contribution to the total number of S9 transcripts was limiting. Lastly, deletion of both introns increased the total number of transcripts encoding S9 to 170% of wild-type levels (Figure 2E, fourth column). Taken together, these data suggest that the *RPS9A* and *RPS9B* genes reciprocally cross-regulate by a common intron-dependent mechanism. Further, the large relative effects detected for *RPS9A* compared to *RPS9B* may simply reflect the large difference in expression level between the two gene copies.

### *Drosophila RpS9* autoregulates through alternative splicing and NMD

Reminiscent of the cross-regulation between *S. cerevisiae RPS9A* and *RPS9B* genes, several metazoan RPGs have been shown to autoregulate through alternative splicing coupled to NMD (so-called “Regulated Unproductive Splicing and Translation” or RUST): a process in which the synthesis of productively-spliced mRNA is repressed in favor of unproductive mRNA isoforms encoding premature termination codons (PTC+) [20]–[23] (reviewed in [24]). While this process is conserved between distantly related eukaryotes, there is no known overlap between the genes regulated by RUST in yeast and metazoans to facilitate mechanistic comparisons. Intriguingly, an alternatively-spliced *RpS9* PTC+ mRNA isoform was recently identified in *Drosophila melanogaster* [25]. Thus, we considered the possibility that other *RPS9* orthologs autoregulate in a manner analogous to *RPS9A* and *RPS9B* cross-regulation.

**Figure 3:**
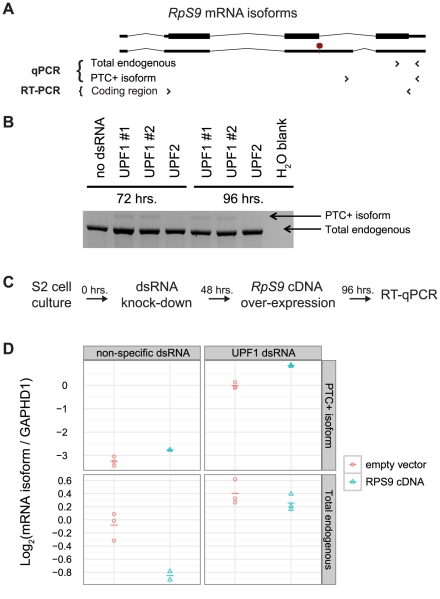
*D. melanogaster RpS9* is autoregulated by alternative splicing coupled to NMD. A) Illustration of *RpS9* mRNA isoforms assessed by PCR (the PTC+ isoform is indicated by a red octagon). Primers sets (arrows) were designed to amplify multiple or specific *RpS9* mRNA isoforms (RT-PCR and qPCR primers, respectively). B) RT-PCR validation of the *RpS9* PTC+ mRNA isoform degraded by NMD. C) Experimental design used to assess the affect of *UPF1* knock-down on the abundance of *RpS9* mRNA isoforms. D) RT-qPCR determination of *RpS9* PTC+ mRNA isoform abundance (top panels) and total endogenous *RpS9* mRNA abundance (bottom panels) in S2 cells transfected with a plasmid constitutively expressing an *RpS9* cDNA (red circles) or an empty vector control (blue circles). The affect of *UPF1* knock-down (via incubation with dsRNA) on each *RpS9* mRNA isoform (right panels) is compared to a non-specific dsRNA control (left panels). *RpS9* mRNA isoform abundance values were divided by *GAPDH1* mRNA abundance values to obtain ratios internally controlled for variations in cDNA quantity. Log_2_ transformed ratios for each of three biological replicates is shown as a point and the mean as a dash.

We hypothesized that *D. melanogaster RpS9* expression is regulated in response to excess protein production by alternative splicing coupled to NMD. Therefore, we predicted that increased *RpS9* expression would result in increased abundance of the PTC+ mRNA isoform. To test this hypothesis, we measured the affect of exogenous *RpS9* overexpression and NMD inhibition on alternative splicing of *RpS9* messages using RT-qPCR primer sets specific to endogenous *RpS9* mRNA isoforms (Figure 3A). We first verified that the previously identified *RpS9* PTC+ isoform in S2 cells was degraded by NMD through RT-PCR amplification of *RpS9* transcripts from S2 cells incubated with either of two dsRNAs targeting *Upf1* (Figure 3B). To then test the effect of increased *RpS9* expression on the abundance of the PTC+ mRNA isoform, we exogenously overexpressed a cDNA copy of *RpS9* (Figure 3C). In S2 cells overexpressing *RpS9*, we detected an increase in the abundance of the PTC-containing mRNA isoform (Figure 3D, top panels, compare red and blue points) and a decrease in the total *RpS9* expression as compared to the empty vector control (Figure 3D, bottom left panel, compare red and blue points). As expected, we observed a UPF1-dependent decrease in total endogenous *RpS9* abundance in response to increased *RpS9* expression (Figure 3D, compare bottom left and right panels, blue points). Taken together, these results suggests that Drosophila *RpS9* autoregulates by RUST, in which excess expression shifts the balance of alternative splicing from the synthesis of productively spliced messages towards the synthesis of unproductive *RpS9* PTC+ messages that are selectively degraded by NMD.

### Diverse forms of *RPS9* alternative splicing are associated with structured and conserved RNA sequences

**Figure 4:**
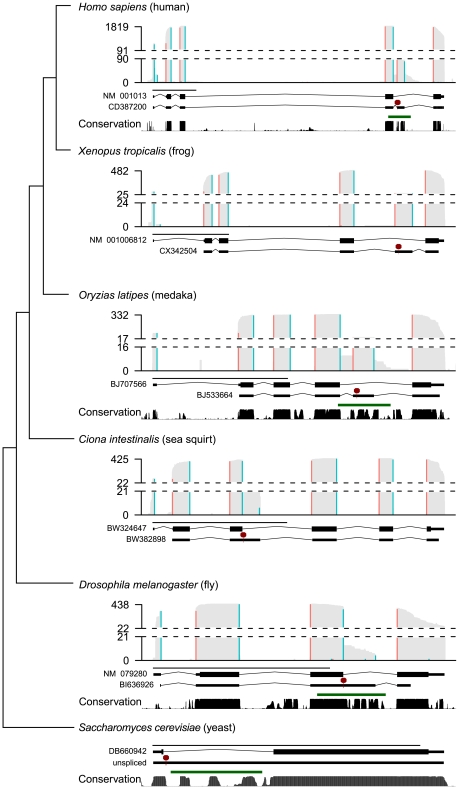
Diverse alternatively spliced *RPS9* isoforms encode PTC+ exons associated with high nucleotide conservation and predicted RNA structures. Summaries of ESTs, predicted RNA structures, and sequence conservation from animal *RPS9* orthologs (*H. sapiens*, *X. tropicalis*, *O. latipes*, *D. melanogaster*, *C. intestinalis*, and *S. cerevisiae*) are presented along a dendogram illustrating their phylogenetic relationships (not to scale). For each species, histograms summarize EST coverage (gray bars) and inferred splice junctions with both 5′ GT (blue bars) and 3′ AG splice sites (red bars). Dashed lines separate the lower 5% and upper 95% histogram values; EST coverage is labeled on the y-axis. Two gene models (below each histogram) are plotted to scale (black line; 1 kb) representing either the major isoform (top gene model) or a spliced PTC+ EST (bottom gene model) for each species (an “unspliced” pre-mRNA is modeled for S. cerevisiae in lieu of an EST). The major isoform sequence is annotated as coding (thick black lines) or UTR (thin black lines) and interrupted by GT-AG introns (angled black lines). The first PTC (red line and octagon) in the representative PTC+ EST sequence (thin lines) is indicated. Below the two gene models, PhastCons scores (black bars), and RNAz predictions (green lines) indicate regions associated with high nucleotide conservation and statistically significant (P>0.9) RNA structure predictions, respectively (*X. tropicalis* not shown; *C. intestinalis* not applicable). PhastCons scores and RNAz predictions were based on MultiZ alignments obtained from the UCSC Genome Browser where available (see Methods).

We hypothesized that *RpS9* autoregulation had an important function and would thus be conserved in other animals. Further, we hypothesized that conserved RNA structures were involved in the cross-regulation of *RPS9A* and *RPS9B* in *S. cerevisiae* and the autoregulation of *RpS9* in *D. melanogaster*, because *E. coli* S4 (the bacterial ortholog), requires an RNA structure to autoregulate by translational repression. Therefore, we predicted that *RPS9* orthologs would be associated with alternatively-spliced mRNA isoforms, conserved RNA structures, and PTCs. To identify such messages, we summarized expressed sequence tags (ESTs) data from diverse animals. Indeed, EST coverage extends outside exons and into introns, which support the existence of rare unspliced or alternatively-spliced transcripts (<5% maximum coverage) (Figure 4, gray bars). To identify ESTs that specifically support alternative splice site usage or cassette exon inclusion, we mapped putative EST exon-exon junctions that spanned both 5′ GT and 3′ AG splice sites (Figure 4, blue and red bars, respectively). With the exception of *Petromyzon marinus*, ESTs from various vertebrates (e.g. *H. sapiens*, *Rattus norvegicus*, *Xenopus tropicalis*, *Danio rerio*, and *Oryzias latipes*) reveal cassette exons that introduce PTCs from the last canonical intron (Figure 4 and Figure S1). *P. marinus* and *D. melanogaster* ESTs, on the other hand, reveal alternative 5′ splice sites that also introduce PTCs from a homologous intron (Figure 4 and Figure S1). Most intriguingly, *Ciona intestinalis* ESTs also support alternative 5′ splice site usage, but in a non-homologous intron compared to those of other animals (Figure 4). Thus, our surveys of animal ESTs suggest that animal *RPS9* orthologs are often alternatively-spliced to utilize RUST. Further, the conservation of alternatively-spliced cassette exons within the last intron among distantly related vertebrates (e.g. ∼400 million years between humans and fish [10]) suggest that these isoforms are functional.

Also consistent with function, PTC positions in *RPS9* orthologs were associated with high nucleotide conservation (Figure 4). To determine if *RPS9* orthologs were also associated with thermodynamically-stable and structurally-conserved RNA structures, we screened the gene bodies of *RPS9* orthologs for statistically significant RNA structures using RNAz [26] on alignments obtained from the UCSC Genome Browser [27]. In order to examine both intronic and exonic sequences, we obtained sets of nucleotide alignments from closely-related groups of organisms: mammals, drosophilids, teleosts, and hemiascomycetous yeasts. Scanning *RPS9* ortholog alignments in 400 bp windows, we identified predicted RNA structures (P>0.9), specifically within the last intron of mammalian, drosophilid, and teleost *RPS9* orthologs, each overlapping with PTC positions (Figure 4, green lines and red octagons). Similarly, sequence alignments of *RPS9* orthologs from hemiascomycetous yeasts also revealed predicted RNA structures specifically within the single yeast intron, which if unspliced, would introduce a PTC (Figure 4). Due to the lack of sequences similar to the *C. intestinalis RPS9* gene corresponding to the PTC in its third intron, we did not test this region for conserved elements and predicted RNA structures. In any case, these data indicate the potential for autoregulation among distantly related *RPS9* orthologs through the use of different forms of alternative splicing, perhaps through structured RNA elements.

## Discussion

The complex evolutionary history of introns immediately raises three fundamental questions. First, why do introns persist? Second, what functions of introns promote their selection and persistence? Third, are intron functions general across species, or have they acquired different functions in different organisms? Our study sheds light onto these questions.

### Biased intron loss may reflect selection for functional introns

The genes of *S. cerevisiae*, and hemiascomycetous yeasts in general, contain very few introns compared to other eukaryotes [28], which is generally attributed to uncommonly high rates of intron loss within this lineage [15]. Previous observations that *S. cerevisiae* introns are biased for RPGs [1], [2], [29], [30] and other highly expressed genes [4] have been cited as evidence that many *S. cerevisiae* RPG introns have one or more functions. Intriguingly, similar biases are also observed in the intron-poor genomes of *Encephalitozoon cuniculi* [31], [32] and the nucleomorph of *Guillardia theta* [33], suggesting that the bias against RPG intron loss is not limited to yeasts. By measuring the rates of intron loss among recently-duplicated genes, we confirm that an ongoing bias against RPG intron loss is apparent in the lineage leading to *S. cerevisiae* (Figure 1). Thus, the few remaining introns in *S. cerevisiae* may reflect biases in 1) the mechanisms of intron loss and/or 2) selection to keep important introns. In addition to previously-proposed functions of RPG introns (see below), several lines of evidence suggest that the conservation of RPG introns is not merely a function of mutation rates. Reverse transcription-mediated intron loss is expected to preferentially remove 3′ end biased introns from highly-expressed genes [34]. First, intron biases for RPGs run counter to the expectation for intron loss among highly-expressed genes, since these transcripts would be more likely to be reverse transcribed (as discussed in [32]). Second, the majority of *S. cerevisiae* intron losses observed here are not 3′ end biased; in fact, several introns were lost from the 5′ UTR (e.g. *GBP1*, *NHP6A* and *ARF1*). Lastly, at least 21 RPG introns that are present in both the *Lachantea* and *Saccharomyces* clades appear to have been lost from *Z. rouxii*, indicating that species-specific RPG intron losses can occur, but have not done so in the lineage leading to *S. cerevisiae* (Table S1). Biased intron loss, therefore, may reflect species-specific selective pressure to retain functional introns.

### Intron function in the absence of alternative spicing

In many eukaryotes, the presence of large numbers of introns permit alternative splicing, which can be used to increase protein diversity [35]. However, the simple gene architectures of *S. cerevisiae* provide limited opportunity for the generation of multiple protein isoforms through alternative splicing (although a few instances have been described [36], [37]). Instead, *S. cerevisiae* RPG introns have been proposed to confer other functions, such as transcriptional enhancement [4] and splicing regulation. We tested these two hypotheses directly by deleting introns from 16 *S. cerevisiae* genes and assessing the effect on gene expression by microarray.

Unlike intronless copies of some mammalian genes [38], the expression of many RPGs were unaffected or even increased by deleting introns (Figure 2A). Thus, the persistence of these introns may be due to selection for other intron functions, such as splicing regulation, perhaps in response to amino acid starvation [16]. Alternatively, our splicing microarray platform may not provide the sensitivity needed to confidently identify subtle, but potentially important, changes in expression levels. Nonetheless, we did observe large increases in gene expression for *RPS14B*, which is known to autoregulate through splicing inhibition [7]. Thus, it seems likely that this intron and the *RPS9A* and *RPS9B* introns are under additional selection pressure to maintain homeostasis of protein levels. Consistent with this view, regions within the *RPS9A* and *RPS9B* introns are highly conserved (Figure 4 and Figure S2), which strongly suggests that mutations within these introns have been detrimental to fitness during natural history. Therefore, the strong bias against RPG intron loss (see above) may reflect ongoing selection for splicing regulation.

### Evolution of the *RPS9* autoregulatory circuit

The propensity for RNA-binding proteins to utilize alternative splicing for the purpose of autoregulation has long been noted [39] and, in the case of RNA-binding proteins, is remarkably common [40]–[42]. To our knowledge, however, regulation at the level of splicing between organisms as evolutionarily distant as *S. cerevisiae* and humans is exceedingly rare. While autoregulation of RPGs by alternative splicing is common and can be conserved as distantly as worms and humans [20], [21], we find no evidence that other yeast RPGs (i.e. *RPL30* and *RPS14*) are regulated by splicing in both yeast and mammals (Figure S3). Interestingly, S9 orthologs in bacteria (and possibly archaea) are among a small class of RPGs that autoregulate by translational repression [43], [44]. Thus, an intriguing notion is that S9 autoregulation is of particular importance to life or particularly likely to evolve. Presumably, autoregulation of S9 production would benefit the cell by reducing waste [3] and by preventing potentially harmful interactions with low-affinity targets [45].

**Figure 5:**
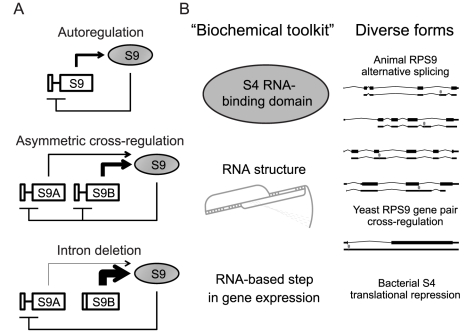
Hypothetical evolution of *RPS9* autoregulation. A) Hypothetical evolution of the *RPS9* autoregulatory circuit after duplication and divergence. Autoregulation of pre-WGD *RPS9* (top) is conserved between post-WGD gene copies despite divergence in expression levels to produce asymmetrical cross-regulation (middle). In *S. cerevisiae*, *RPS9A* and *RPS9B* intron deletions shift the burden of autoregulation onto the other intron-containing gene copy (bottom). B) A theoretical “biochemical toolkit,” which minimally requires an S4 RNA-binding domain and a suitable RNA binding site to perturb an essential step in gene expression (left), could potentially produce the many forms of splicing regulation observed in yeasts and animal *RPS9* orthologs (right).

Cross-regulation, such as between the *RPS9A* and *RPS9B* genes, has also been observed between multiple sets of paralogous splicing regulators, including hnRNPL and hnRNPLL [46], as well as PTB, nPTB and ROD1 [47]. We speculate that these genes exemplify a straightforward principle of gene duplication and evolution: upon gene duplication, autoregulation would inherently become cross-regulation. As the paralogs diverge in abundance and/or protein function, this cross-regulation could become asymmetric (Figure 5A). In theory, such asymmetric cross-regulation among RPG pairs may allow differential expression of functionally-distinct ribosomal proteins to produce a “ribosome code” [48]. What distinct functions are provided by *RPS9A* and *RPS9B* gene products remain to be seen. Interestingly, the *RPS9A* and *RPS9B* genes encode S9 proteins that differ primarily within a small C-terminal acidic patch that may be required for proper ribosomal disassociation [49]. Mutational analyses of these three differing amino acids are needed to definitively test whether *S. cerevisiae* utilizes differential expression of *RPS9A* and *RPS9B* genes to exploit functional differences in the proteins they encode.

How does excess S9 regulate the splicing of the *RPS9* orthologs? One possibility is that S9 binds its own mRNA like bacterial S4. A strong paradigm has been set by S14 and L30 in yeast and S26 and S13 in animals, in which these ribosomal proteins bind RNA structures present in their introns [6], [7], [22], [23]. It seems likely that S9 might operate under the same paradigm. Intuitively, conserved RNA structures within the introns of *RPS9* orthologs make for likely targets for S9 binding (Figure 4). However, we do not observe obvious similarities between these predicted structures and the *E. coli* S4 regulatory site, which forms a double pseudoknot [43]. *E. coli* S4 can also bind and regulate a *Bacillis subtilis* mRNA that contains a dissimilar pseudoknot structure [50]. Intriguingly, the conserved RNA structures in *RPS9A* and *RPS9B* also appear to have the potential to form a pseudoknot (Figure S4). Thus, it seems plausible that the putative RNA structures within yeast and animal introns may yet be binding sites for S9 despite considerable structural divergence. This, however, is mere speculation and *in vitro* binding assays are needed to determine if ribosomal protein S9 directly regulates its own expression in *S. cerevisiae* and other eukaryotes. If auto- and cross-regulation were indeed directly mediated by ribosomal protein S9 binding, then comparative biochemical studies using proteins and RNA sequences from different species could provide mechanistic detail to describe how S9 mediates the different forms of alternative splicing.

Why are there so many forms of splicing regulation among *RPS9* orthologs? One possibility is that particular aspects of these forms are ancient and conserved, while others have evolved independently in different lineages. For example, the genetic circuits that specify the development of diverse animal forms (e.g. eyes and limbs) exemplify deep homology, where recent evolutionary innovations overlay a shared “genetic toolkit” [51]. By analogy, genetic circuits themselves (in this case, autoregulation) may share a common “biochemical toolkit” comprised of highly conserved biochemical processes (e.g. RNA∶protein interactions), while independently evolving elaborations on these basic circuits. Thus, translational inhibition of the alpha-operon by S4-binding may represent just one of many possible forms of regulation accessible to the highly conserved S4 RNA-binding domain proteins found throughout cellular life. Alternative splicing in animals and regulated splicing in *S. cerevisiae* may be different elaborations on this autoregulatory circuit, perhaps mediated by different RNA structures within introns (Figure 5B). Thus, we propose that the highly-conserved function of ribosomal protein S9 (and RNA-binding proteins in general) is one part of a biochemical toolkit that is frequently used and reused, as the fundamental autoregulatory circuit is maintained, elaborated and reinvented.

## Methods

### Intron gain and loss analysis

To estimate the propensity for intron loss among RPGs and non-RPGs, we compared annotated *S. cerevisiae* intron-containing genes and WGD-derived gene pairs. *S. cerevisiae* intron annotations were obtained from the *Saccharomyces* Genome Database (http://www.yeastgenome.org/) on 7/20/2011. WGD-derived gene pairs (as inferred from genomic synteny; a.k.a. “Ohnologs”) were obtained from the Yeast Gene Order Browser (http://wolfe.gen.tcd.ie/ygob/) [14]. Because introns are commonly identified by gaps in BLAST-based homology searches, intron-containing genes with short first exons are commonly misannotated. To identify annotated introns upstream of an annotated gene, custom scripts written in R (http://www.r-project.org/) were used to scan 800 bp upstream and 100 bp downstream of the ORF start site with a regular expression that recognizes >90% of *S. cerevisiae* introns by identifying the most common splice sites to minimize false positive matches. The regular expression matches sequences meeting the following criteria in order: 1) any one of the 4 most common of 5′ SSs, 2) an S1 length of at least 30, 3) any one of the 5 most common branchsites, 4) an S2 length between 1 and 50, and 5) any one of the 3 most common 3′ SS trinucleotides, which was formalized as: “(gtatgt|gtacgt|gtaagt|gtatga).{30,}?(tactaac|gactaac|aactaac|tgctaac|cactaac).{1,50}?[tca]ag”. The pre-WGD ancestor was inferred to contain an intron if the majority of available outgroup pre-WGD species orthologs (*C. albicans, L. waltii, L. thermotolerans, L. kluyveri, E. gossypii, K. lactis*) and 1) the *Z. rouxii* ortholog had an intron or 2) the majority of post-WGD species intron (*S. cerevisiae*, *S. bayanus*, *C. glabrata*, *N. castellii* and *V. polyspora*) gene pairs had at least one intron. In this manner, we distinguished independent intron gains and losses in *Z. rouxii* from intron gains and losses immediately after the WGD event.

### *S. cerevisiae* strain construction

Intron deletion mutants were generated by a replacement strategy similar to a previously-described method for intron deletion [52]. Briefly, a PCR product amplified from the plasmid pJPS1232 (generously provided by J. Staley, University of Chicago), which contains the CORE construct [53] fused to the I-SceI endonuclease site, using gene-specific primers containing exon 1 and exon 2 sequences that allow integration and subsequent intron deletion via homologous recombination. Transformed diploid cells (yAP047) were incubated for 4 h at 30°C in the presence of 2% galactose to induce I-SceI endonuclease expression and precise deletion of the CORE cassette. Sporulated haploid cells were confirmed to harbor intron deletions by PCR. To ensure that a precise intron deletion was obtained without any additional mutations, the region surrounding the newly-created exon-exon junction (at least 100 bp) was PCR amplified and sequenced. Strains described in Figure 2 were also confirmed for Δi mutation by decreased microarray intron probe signal. Gene specific primers used for mutagenesis are detailed in Table S2.

### *D. melanogaster* S2 cell RNAi and transfection

Routine passaging of S2 cell cultures and RNAi depletion was performed as described [54] with the following modifications. Briefly, 1 µg/ml of dsRNA was incubated with 3.5E5 cells in 350 µl of media in 24-well plates. After 48 h incubation with dsRNA, cells were transfected with 0.2 µg plasmid with Effectene (Quigen) according to manufacturer's instructions. Cells were harvested with 1 ml TRIzol (Invitrogen) for analysis by RT-qPCR (see below). Primers used to generate PCR products used for dsRNA synthesis (Promega RiboMAX) are described in Table S2.

### RNA isolation and cDNA synthesis

To analyze the expression of genes in *S. cerevisiae* intron deletion strains, 15 ml cultures of mutant and wild-type yeast were grown in parallel at 30°C in rich medium supplemented with 2% glucose to an optical density between A_600_ = 0.5 and 0.7. For microarray hybridization, RNA was isolated by acid-phenol extraction and converted to cDNA as described [16]. A similar protocol was performed for qPCR applications with the following modifications. After RNA isolation, 2 µg of DNase-treated RNA was random primed in a 40 µl reaction containing 1 µg dN9 primer, 50 mM TrisHCl (pH 8.4), 75 mM KCl, 3 mM MgCl2, 10 mM DTT, 0.5 mM dNTPs, and 5 ng murine Moloney leukemia virus (M-MLV) RT. Primers were hybridized at 60°C for 7 min prior to the addition of enzyme, and then incubated with enzyme at 42°C for at least 2 h. Prepared cDNA was diluted at least 10-fold before use in qPCR. Similarly, to analyze *D. melanogaster* S2 cell, 725 µl cultures of S2 cells (UCSF cell culture facility) were grown in 24-well plates at 25°C in Schneider's Drosophila Medium (Gibco) supplemented with 10% fetal bovine serum (UCSF) to a count of ∼5E6 cells. RNA was extracted with 1 ml TriZol (Invitrogen) according to manufacturer's instructions. After RNA isolation, cDNA was prepared with SuperScript III (Invitogen) and random priming according to manufacturer's instructions. Prepared cDNA was diluted at least 10-fold before use in qPCR.

### Microarray analysis

Splicing-sensitive microarrays were constructed and performed as described [16]. In each experiment, a wild-type strain derived from the same parent as the intron deletion mutant strain was used as a reference. Data was analyzed using the R Bioconductor packages marray() and limma() [55] in a custom pipeline based on the Goulphar program [56]. Microarray data used in this study are available in the Gene Expression Omnibus at NCBI (GSE35541).

### Quantitative PCR

Quantitative PCR primers (Table S2) were designed using Primer3 [57] and *S. cerevisiae* or *D. melanogaster* genomic sequence obtained from the UCSC Genome Browser (SacCer1 or dm3, respectively) [58], [59]. Serial dilutions of DNA ranging from 100 to 0.16 ng of the genomic DNA were used to obtain calibration curves, measure primer efficiencies, and ensure that quantification was in a linear dynamic range. Primer sets yielding multiple amplification products or calibration curves with R-squared values of <0.96 were excluded. For each qPCR sample, diluted cDNA was amplified in 25 µl volume reactions containing 250 µM dNTPs, 1× (NH_4_)_2_SO_4_ buffer (Fermentas), 0.5 µM primer, 1.5 mM MgCl2, 1.25 Units Dynazyme II (Finnzymes), and Sybr Green I fluorescent dye (Sigma). Fluorescence was measured on a BioRad Opticon machine using standard cycling conditions (3 min at 95°C, 40 cycles of 15 s at 95°C, 30 s at 55°C, and 15 s at 72°C). Biological replicate qPCR values were determined as the median of technical replicates. For each of 3 biological replicates, target gene values (e.g. *RPS9A*) were divided by reference gene values (e.g. *SCR1*) before log transformation. Plots were generated using the R package ggplot2() [60].

### Assessment of alternative splicing among animal *RPS9* orthologs

To assess EST coverage and splicing, we obtained genomic coordinates corresponding to GenBank ESTs from the UCSC Genome Browser ‘spliced EST’ track, which span at least one canonical intron of at least 32 bases [61]. Custom R scripts were used to calculate EST coverage per genomic nucleotide position, and identify all exon-exon junctions that span putative GT/AG splice site [62]. The following genome assemblies were used in the analysis: *Branchiostoma floridae* (braFlo1); *Ciona intestinalis* (Ci2); *Danio rerio* (danRer7); *Drosophila melanogaster* (dm3), *Oryzias latipes* (oryLat2); *Petromyzon marinus* (petMar1); *Xenopus tropicalis* (xenTro2); *Rattus norvegicus* (rn4); *Mus musculus* (mm9); *Homo sapiens* (hg19).

## Supporting Information

Figure S1 Comparison of spliced isoforms by EST analysis of *RPS9* orthologs from 10 animals. EST summaries of *RPS9* orthologs from 10 animal species illustrated as in Figure 4. Genes are plotted to scale (black line; 1 kb).
(PDF)

Figure S2 Conserved intronic regions among yeast *RPS9* orthologs. Nucleotide alignment of genes encoding ribosomal protein S9 from *L. waltii*, *L. thermotolerans*, *L. kluyveri*, *E. gossypii*, *K. lactis*, *Z. rouxii*, *S. cerevisiae*, *S. bayanus*, *C. glabrata*, *N. castellii* and *V. polyspora*. Note that all genes contain an intron with regions of identical sequences (black shading). Positions of compensatory base pair changes supporting RNA stems shown in Figure S4 are highlighted in purple.
(PDF)

Figure S3 EST analysis of mammalian *RPL30* and *RPS14* does not reveal conserved alternatively-spliced isoforms. EST summaries of *RPL30* and *RPS14* orthologs from human, mouse, and rat illustrated as in Figure 4. Genes are plotted to scale (black line; 1 kb).
(PDF)

Figure S4 Putative RNA structure within the *RPS9A* and *RPS9B* introns. Conserved elements in the *RPS9A* and *RPS9B* introns are associated with a putative pseudoknot structure near the 5′ splice site. A) An illustration of the *S. cerevisiae RPS9A* gene model and nucleotide conservation among closely related yeasts (from the UCSC Genome Browser). Putative RNA stems (predicted by the RNAz program [26]) that overlap with conserved regions are indicated (numbered 1–4). B) Illustration of a putative H-H type pseudoknot (predicted by the IPknot program [64]) based on nucleotide alignment of pre- and post-WGD yeasts (Figure S2). Positions of compensatory base pair changes that support the pseudoknot stems are indicated in purple. Pseudoknot illustration was created with PseudoViewer v3.0 (http://pseudoviewer.inha.ac.kr/).
(PDF)

Table S1 Intron annotations and predictions.
(PDF)

Table S2 Primers.
(PDF)

## References

[1] Bon E, Casaregola S, Blandin G, Llorente B, Neuvéglise C (2003). Molecular evolution of eukaryotic genomes: hemiascomycetous yeast spliceosomal introns.. Nucleic Acids Res.

[2] Mitrovich QM, Tuch BB, Guthrie C, Johnson AD (2007). Computational and experimental approaches double the number of known introns in the pathogenic yeast Candida albicans.. Genome Res.

[3] Warner JR (1999). The economics of ribosome biosynthesis in yeast.. Trends Biochem Sci.

[4] Juneau K, Miranda M, Hillenmeyer ME, Nislow C, Davis RW (2006). Introns regulate RNA and protein abundance in yeast.. Genetics.

[5] Pleiss JA, Whitworth GB, Bergkessel M, Guthrie C (2007). Rapid, transcript-specific changes in splicing in response to environmental stress.. Mol Cell.

[6] Dabeva MD, Warner JR (1993). Ribosomal protein L32 of Saccharomyces cerevisiae regulates both splicing and translation of its own transcript.. J Biol Chem.

[7] Fewell SW, Woolford JL (1999). Ribosomal protein S14 of Saccharomyces cerevisiae regulates its expression by binding to RPS14B pre-mRNA and to 18S rRNA.. Mol Cell Biol.

[8] Dean D, Nomura M (1980). Feedback regulation of ribosomal protein gene expression in Escherichia coli.. Proc Natl Acad Sci USA.

[9] Nomura M, Gourse R, Baughman G (1984). Regulation of the synthesis of ribosomes and ribosomal components.. Annu Rev Biochem.

[10] Hedges SB, Dudley J, Kumar S (2006). TimeTree: a public knowledge-base of divergence times among organisms.. Bioinformatics.

[11] Wolfe KH, Shields DC (1997). Molecular evidence for an ancient duplication of the entire yeast genome.. Nature.

[12] Juneau K, Palm C, Miranda M, Davis RW (2007). High-density yeast-tiling array reveals previously undiscovered introns and extensive regulation of meiotic splicing.. Proc Natl Acad Sci USA.

[13] Zhang Z, Hesselberth JR, Fields S (2007). Genome-wide identification of spliced introns using a tiling microarray.. Genome Res.

[14] Byrne KP, Wolfe KH (2005). The Yeast Gene Order Browser: combining curated homology and syntenic context reveals gene fate in polyploid species.. Genome Res.

[15] Stajich JE, Dietrich FS, Roy SW (2007). Comparative genomic analysis of fungal genomes reveals intron-rich ancestors.. Genome Biol.

[16] Pleiss JA, Whitworth GB, Bergkessel M, Guthrie C (2007). Transcript specificity in yeast pre-mRNA splicing revealed by mutations in core spliceosomal components.. PLoS Biol.

[17] Li Z, Paulovich AG, Woolford JL (1995). Feedback inhibition of the yeast ribosomal protein gene CRY2 is mediated by the nucleotide sequence and secondary structure of CRY2 pre-mRNA.. Mol Cell Biol.

[18] Clark TA, Sugnet CW, Ares M (2002). Genomewide analysis of mRNA processing in yeast using splicing-specific microarrays.. Science.

[19] Nagalakshmi U, Wang Z, Waern K, Shou C, Raha D (2008). The transcriptional landscape of the yeast genome defined by RNA sequencing.. Science.

[20] Mitrovich QM, Anderson P (2000). Unproductively spliced ribosomal protein mRNAs are natural targets of mRNA surveillance in C. elegans.. Genes Dev.

[21] Cuccurese M, Russo G, Russo A, Pietropaolo C (2005). Alternative splicing and nonsense-mediated mRNA decay regulate mammalian ribosomal gene expression.. Nucleic Acids Res.

[22] Ivanov AV, Malygin AA, Karpova GG (2005). Human ribosomal protein S26 suppresses the splicing of its pre-mRNA.. Biochim Biophys Acta.

[23] Malygin AA, Parakhnevitch NM, Ivanov AV, Eperon IC, Karpova GG (2007). Human ribosomal protein S13 regulates expression of its own gene at the splicing step by a feedback mechanism.. Nucleic Acids Res.

[24] Lareau LF, Brooks AN, Soergel DAW, Meng Q, Brenner SE (2007). The coupling of alternative splicing and nonsense-mediated mRNA decay.. Adv Exp Med Biol.

[25] Hansen KD, Lareau LF, Blanchette M, Green RE, Meng Q (2009). Genome-wide identification of alternative splice forms down-regulated by nonsense-mediated mRNA decay in Drosophila.. PLoS Genet.

[26] Washietl S, Hofacker IL, Stadler PF (2005). Fast and reliable prediction of noncoding RNAs.. Proc Natl Acad Sci USA.

[27] Kent WJ, Sugnet CW, Furey TS, Roskin KM, Pringle TH (2002). The human genome browser at UCSC.. Genome Res.

[28] Jeffares DC, Mourier T, Penny D (2006). The biology of intron gain and loss.. Trends Genet.

[29] Ares M, Grate L, Pauling MH (1999). A handful of intron-containing genes produces the lion's share of yeast mRNA.. RNA.

[30] Spingola M, Grate L, Haussler D, Ares M (1999). Genome-wide bioinformatic and molecular analysis of introns in Saccharomyces cerevisiae.. RNA.

[31] Katinka MD, Duprat S, Cornillot E, Méténier G, Thomarat F (2001). Genome sequence and gene compaction of the eukaryote parasite Encephalitozoon cuniculi.. Nature.

[32] Lee RCH, Gill EE, Roy SW, Fast NM (2010). Constrained intron structures in a microsporidian.. Mol Biol Evol.

[33] Douglas S, Zauner S, Fraunholz M, Beaton M, Penny S (2001). The highly reduced genome of an enslaved algal nucleus.. Nature.

[34] Fink GR (1987). Pseudogenes in yeast?. Cell.

[35] Nilsen TW, Graveley BR (2010). Expansion of the eukaryotic proteome by alternative splicing.. Nature.

[36] Grund SE, Fischer T, Cabal GG, Antúnez O, Pérez-Ortín JE (2008). The inner nuclear membrane protein Src1 associates with subtelomeric genes and alters their regulated gene expression.. J Cell Biol.

[37] Juneau K, Nislow C, Davis RW (2009). Alternative splicing of PTC7 in Saccharomyces cerevisiae determines protein localization.. Genetics.

[38] Brinster RL, Allen JM, Behringer RR, Gelinas RE, Palmiter RD (1988). Introns increase transcriptional efficiency in transgenic mice.. Proc Natl Acad Sci USA.

[39] Mattox W, Ryner L, Baker BS (1992). Autoregulation and multifunctionality among trans-acting factors that regulate alternative pre-mRNA processing.. J Biol Chem.

[40] Lareau LF, Inada M, Green RE, Wengrod JC, Brenner SE (2007). Unproductive splicing of SR genes associated with highly conserved and ultraconserved DNA elements.. Nature.

[41] Ni JZ, Grate L, Donohue JP, Preston C, Nobida N (2007). Ultraconserved elements are associated with homeostatic control of splicing regulators by alternative splicing and nonsense-mediated decay.. Genes Dev.

[42] Saltzman AL, Kim YK, Pan Q, Fagnani MM, Maquat LE (2008). Regulation of multiple core spliceosomal proteins by alternative splicing-coupled nonsense-mediated mRNA decay.. Mol Cell Biol.

[43] Tang CK, Draper DE (1989). Unusual mRNA pseudoknot structure is recognized by a protein translational repressor.. Cell.

[44] Sano K, Taguchi A, Furumoto H, Uda T, Itoh T (1999). Cloning, sequencing, and characterization of ribosomal protein and RNA polymerase genes from the region analogous to the alpha-operon of escherichia coli in halophilic archaea, halobacterium halobium.. Biochem Biophys Res Commun.

[45] Von Hippel PH, Kowalczykowski SC, Lonberg N, Newport JW, Paul LS (1982). Autoregulation of gene expression. Quantitative evaluation of the expression and function of the bacteriophage T4 gene 32 (single-stranded DNA binding) protein system.. J Mol Biol.

[46] Rossbach O, Hung L-H, Schreiner S, Grishina I, Heiner M (2009). Auto- and Cross-Regulation of the hnRNP L Proteins by Alternative Splicing.. Mol Cell Biol.

[47] Spellman R, Llorian M, Smith CWJ (2007). Crossregulation and functional redundancy between the splicing regulator PTB and its paralogs nPTB and ROD1.. Mol Cell.

[48] Komili S, Farny NG, Roth FP, Silver PA (2007). Functional specificity among ribosomal proteins regulates gene expression.. Cell.

[49] Pnueli L, Arava Y (2007). Genome-wide polysomal analysis of a yeast strain with mutated ribosomal protein S9.. BMC Genomics.

[50] Grundy FJ, Henkin TM (1991). The rpsD gene, encoding ribosomal protein S4, is autogenously regulated in Bacillus subtilis.. J Bacteriol.

[51] Shubin N, Tabin C, Carroll S (2009). Deep homology and the origins of evolutionary novelty.. Nature.

[52] Parenteau J, Durand M, Véronneau S, Lacombe A-A, Morin G (2008). Deletion of many yeast introns reveals a minority of genes that require splicing for function.. Mol Biol Cell.

[53] Storici F, Durham CL, Gordenin DA, Resnick MA (2003). Chromosomal site-specific double-strand breaks are efficiently targeted for repair by oligonucleotides in yeast.. Proc Natl Acad Sci USA.

[54] Rogers SL, Rogers GC (2008). Culture of Drosophila S2 cells and their use for RNAi-mediated loss-of-function studies and immunofluorescence microscopy.. Nat Protoc.

[55] Gentleman RC, Carey VJ, Bates DM, Bolstad B, Dettling M (2004). Bioconductor: open software development for computational biology and bioinformatics.. Genome Biol.

[56] Lemoine S, Combes F, Servant N, Le Crom S (2006). Goulphar: rapid access and expertise for standard two-color microarray normalization methods.. BMC Bioinformatics.

[57] Rozen S, Skaletsky H (2000). Primer3 on the WWW for general users and for biologist programmers.. Methods Mol Biol.

[58] Cherry JM, Ball C, Weng S, Juvik G, Schmidt R (1997). Genetic and physical maps of Saccharomyces cerevisiae.. Nature.

[59] Adams MD, Celniker SE, Holt RA, Evans CA, Gocayne JD (2000). The genome sequence of Drosophila melanogaster.. Science.

[60] Wickham H (2009). ggplot2: Elegant Graphics for Data Analysis.

[61] Benson DA, Karsch-Mizrachi I, Lipman DJ, Ostell J, Sayers EW (2011). GenBank.. Nucleic Acids Res.

[62] R Development Core Team (2011). Development Core Team, R: A language and environment for statistical computing.

[63] Hedtke SM, Townsend TM, Hillis DM (2006). Resolution of phylogenetic conflict in large data sets by increased taxon sampling.. Syst Biol.

[64] Sato K, Kato Y, Hamada M, Akutsu T, Asai K (2011). IPknot: fast and accurate prediction of RNA secondary structures with pseudoknots using integer programming.. Bioinformatics.

