# Supplementary material for: Diverse Forms of RPS9 Splicing Are Part of an Evolving Autoregulatory Circuit
Source: PLoS Genet. 2012 Mar 29;8(3):e1002620. doi: 10.1371/journal.pgen.1002620 (PMC3315480; doi:10.1371/journal.pgen.1002620)
Supplement: Figure S1 — Comparison of spliced isoforms by EST analysis of RPS9 orthologs from 10 animals. EST summaries of RPS9 orthologs from 10 animal species illustrated as in Figure 4. Genes are plotted to scale (black line; 1 kb). (PDF) [file pgen.1002620.s001.pdf]

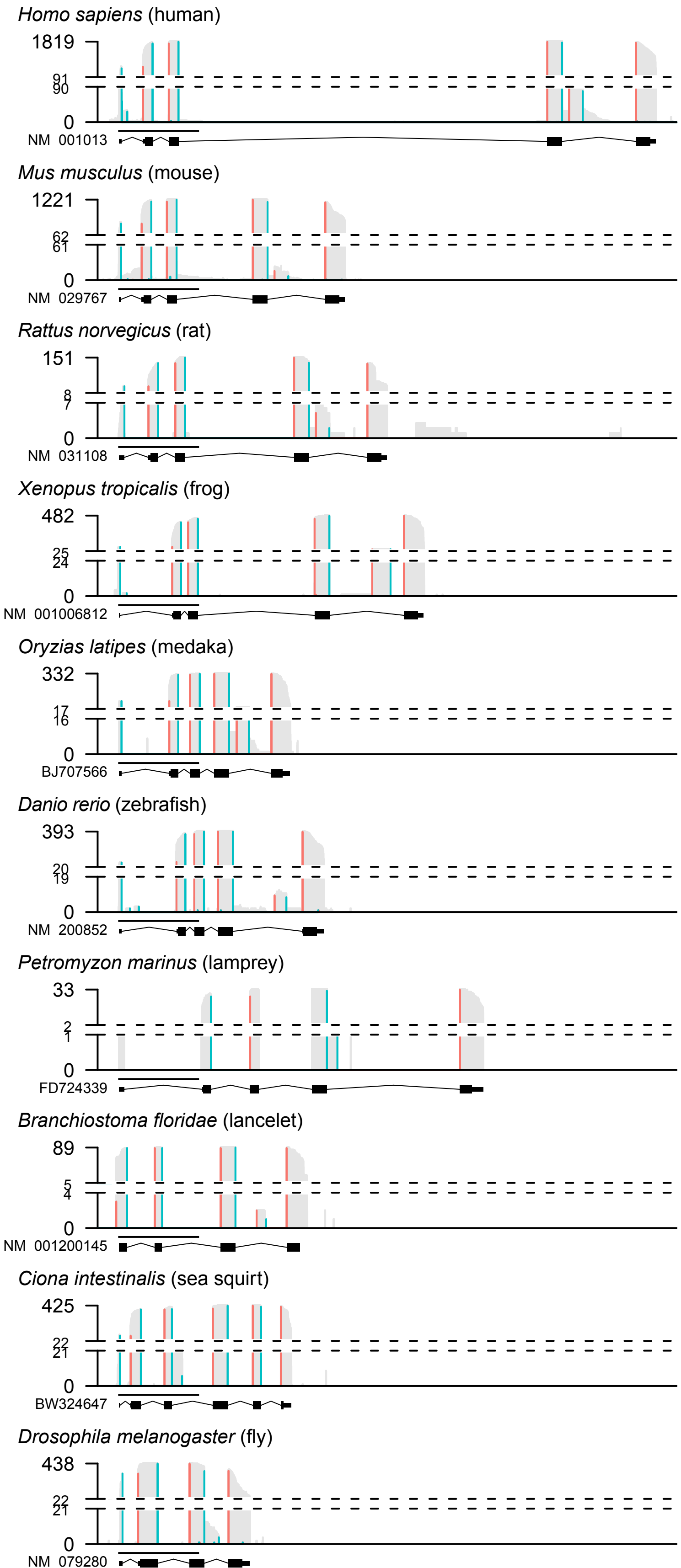

Homo sapiens (human)
1819
91
90
0
NM 001013
Mus musculus (mouse)
1221
62
61
0
NM 029767
Rattus norvegicus (rat)
151
8
7
0
NM 031108
Xenopus tropicalis (frog)
482
25
24
0
NM 001006812
Oryzias latipes (medaka)
332
17
16
0
BJ707566
Danio rerio (zebrafish)
393
20
19
0
NM 200852
Petromyzon marinus (lamprey)
33
2
1
0
FD724339
Branchiostoma floridae (lancelet)
89
5
4
0
NM 001200145
Ciona intestinalis (sea squirt)
425
22
21
0
BW324647
Drosophila melanogaster (fly)
438
22
21
0
NM 079280
