# Supplementary material for: Diverse Forms of RPS9 Splicing Are Part of an Evolving Autoregulatory Circuit
Source: PLoS Genet. 2012 Mar 29;8(3):e1002620. doi: 10.1371/journal.pgen.1002620 (PMC3315480; doi:10.1371/journal.pgen.1002620)
Supplement: Figure S2 — Conserved intronic regions among yeast RPS9 orthologs. Nucleotide alignment of genes encoding ribosomal protein S9 from L. waltii, L. thermotolerans, L. kluyveri, E. gossypii, K. lactis, Z. rouxii, S. cerevisiae, S. bayanus, C. glabrata, N. castellii and V. polyspora. Note that all genes contain an intron with regions of identical sequences (black shading). Positions of compensatory base pair changes supporting RNA stems shown in Figure S4 are highlighted in purple. (PDF) [file pgen.1002620.s002.pdf]

```
Klac_RPS9       1 ATGCCAAGTATGTGGTATCACATTCCAGATATGCTTCATTGATACACTCTGTTACATTTCAAGTCTTTCCTTTGGATTGTCTAGAGATGGGGAAGATAATGCCAGCATCA
Scer_RPS9A      1 ATGCCAAGTACGTCGTTAATC-------------------------------------------------TTCTCCTTTTAAGGGGTTGAGAATATTTTTATTAAAA---
Egos_RPS9       1 ATGCCAAGTACGTA--------------------------------------------------------TATGACATGCGCAGCGAGCGCAGAGCGGCCGGG-------
Kthe_RPS9       1 ATGCCAAGTACGTATTAACATC---------------------ACAACAGATGCCATGCATCCTAACGGATGCAGGCGTCGGAAGAACGAGTTGCACTTGTGC-------
Lwal_RPS9       1 ATGCCAAGTACGTATCAGCATTATGCACAGGTGTCATATCGTTCCATTGGGC------------------GATAAGATGCCGGGAGATGTGGTTACTGTTTAGCTTCTGA
Cgla_RPS9A      1 ATGCCAAGTATGTGCTAATATGGACA-----------------CCACTCTAA------------------AGTGGAATAACTGAAAATGAGAATTGCACTTATATGAGGA
Cgla_RPS9B      1 ATGCCAAGTACGTTACACTAATTTATGATAATAGATAAGCAACACCTGCTTT------------------CTAATAATTTCTAAGCATGATAGTTATCTATCTTTTGCTC
Vpol_RPS9A      1 ATGCCAAGTACGTAT----------------------ATCAAGTTATTCCACTTCA--------------TTCTATATCATACAGATTGATAGTTATGTTTGGTTTTTA-
Vpol_RPS9B      1 ATGCCAAGTATGTTATAATTATTAATT-------------------------------------------TTTCAAAGTATATATATATAAATTGGTATATTTATTATCA
Zrou_RPS9       1 ATGCCAAGTACGTTACAAATATG---------------------------------------------------------------------------------------
Lklu_RPS9       1 ATGCCAAGTACGTATTAACAATAACCACAGACTTACCTTTCCCATGCTCACT------------------TGTGAATTTGGGAAAGGTGGAGATGAGGTCATATCAAT--
Ncas_RPS9A      1 ATGCCAAGTACGTTAT---------------------------CCAATTAAC----------------------------AAACAAAGAAGGATACTATA----------
Ncas_RPS9B      1 ATGCCAAGTACGT-------------------------------------------------------------------------------------------------
Sbay_RPS9A      1 ATGCCAAGTACGTTATTCATC-------------------------------------------------TTATCCTTTTAAAAGGAAAAAGATAAATTTTACGCAATAA
Sbay_RPS9B      1 ATGCCAAGTACGTATTA-----------------------------------------------------CACCCCATCGGGAAAATGAAAGATAGTTCAATCTCTC---
Scer_RPS9B      1 ATGCCAAGTACGTATTA-----------------------------------------------------GACTATATCGAAGAGGAGGGAAATTCTTC-----------
                  Met     5'ss

Klac_RPS9     111 GCT----------------AGCTTAAATCTTC------------------CTTCGAGGA--TCGGGTC---CTCATGATTCAAT-----------TAAAT-----------
Scer_RPS9A     59 -------------------AATCATACTTGCT-------------ATGATTTTGAGGA--TCGGGTC---CTCA--------------------TAAAT----------A
Egos_RPS9      48 -------------------AAACCGGCGCTAG------------------CCTTAGGT--TCGGGTCCTTCGCG------------------------------------
Kthe_RPS9      84 -------------------AATACTCTTCTTT------------------TTCGAGGA--TCGGGTC---CTCA--------------------TGAAT-----------
Lwal_RPS9      93 ACAA---------------AACTCATCTCTTT-------------------TCGAGGA--TCGGGTC---CTCA--------------------TGAAT-----------
Cgla_RPS9A     76 AATGAATTGAGAAATTGAAAAATTGAATAAATGAGAAATTGAAATGAAATATAGAGGTGCTCGGGTCCCTCTCATGAAACGATTTGATTTGATATGAAT-------ACCA
Cgla_RPS9B     93 TACAATTGTAGGGCATTGGACAGGTTATCTTT-------------GAATGCTAGAGGT--TCGGGTC---CTCATTTACAAACTAGATGATACGTTATTTGTTCATCTCA
Vpol_RPS9A     74 -------------------AATAAAAACCTTT--TACATACCTAGTGTATATAGAGGT-TTCGGGTC---CTCATGATCAT-------------TGAAT-----------
Vpol_RPS9B     68 AATCAAATTGGAAATTCTCATTACGATTATCT----------------CCAATGAGGT--TCGGGTCCTCCTTT--------------------TCATT-----------
Zrou_RPS9      24 -------------------AATCAAGACCATT-----------TATGGTCTTAGAGGT--TCGGGTC---CTCA--------------------TAAAT-----------
Lklu_RPS9      91 -------------------GACTCTTTTCTCT------------------TTTGAGGT--TCGGGTC---CTCA--------------------TGAAT-----------
Ncas_RPS9A     46 -------------------AATTGTATTCCTT--------------------CGAGGT--TCGGGTC---CTCA--------------------TAAAT-----------
Ncas_RPS9B     14 -------------------ACCCCAAATCAAG-----------GAATGATTCAGAGGT--TCGGGTC---CTCA--------------------TAAATGATTAATGAGT
Sbay_RPS9A     62 A------------------AGTCATATACACT-----------ATATGACTTTGAGGA--TCGGGTC---CTCA--------------------TAAAT----------A
Sbay_RPS9B     55 -------------------AATCCAATTTCTT---------------TAAGAAGAGGT--TCGGGTC---CTCA--------------------TAAAA----------T
Scer_RPS9B     47 -------------------ACTCTGACTCGTT-----------GATTTTGAAAGAGGT--TCGGGTC---CTCA--------------------TAAAT----------A
                                               Conserved stem-loop 2 (((((   ]]]]]))...)))

Klac_RPS9     161 ---------------------------------TATTGACTCAAAGTATTTCAAGTACTGA---TATTCGATTTGC-AATCTAAACTGTTTCAT-------TTGTTCCT-
Scer_RPS9A    102 TTTTGAAGAGCCTTTGTTGAATTTTT--------TTCTTTTCGTTTTGTTCAATAATGGAC---GGTTCTT--------CATAGAATACTTTTT-------TTTTCGGAG
Egos_RPS9      83 -----------------------------------------------GTTCGCCG----------------------CACTCTGCCCCCACGCC----------------
Kthe_RPS9     121 -----------------------------------------------ATTT----------------------------CTATACCTTCTCAAT-------GTCAAGCCG
Lwal_RPS9     133 -----------------------------------------------ATTT----------------------------CTTCAAACTATCTCT-------GTCAAGCCG
Cgla_RPS9A    179 TTGAGGAATA-------TGAATATACTATCCGCAATATAGCTTACACCTTCACTAAGAGAC---TTGTTTCT-----AGAAGAATGTTTCTGAT-------TACACACTA
Cgla_RPS9B    185 TTTGAGATATCAAATTTTCATCA---------------ATCCTTTTCGTTTGTACA--------ACTTTTTTCTCCTCAGTAAGGATACTCATT-------TATATTTCA
Vpol_RPS9A    135 -------------------------------------TATTCAATACCTTTAAAGATTTTT---TGTTATTTTATCAGATTTGAAACTTGTTTT-------TATGTGTTC
Vpol_RPS9B    129 -----------------------------------------------CTTCATTAAAGAGCAACTT-----------CATTTCAAATTATTTATGATATTGTTGAAATTA
Zrou_RPS9      68 ------------------------------------------------GTTAGAGAATCAA----------------CAATTAAAGCAAGAAAC-------TGGACACAA
Lklu_RPS9     128 -----------------------------------------------ATTCAAAGAA---------TCTGAGAAGAGAATTTCAAATCTTCAAT-------CCAAATTGA
Ncas_RPS9A     81 ------------------------AATAAATTGACAACAAGTCATTTCTTCCATGAGTCAA---TTTCTAAA-----AACTAAAAATACAATCT-------AGAATTTTA
Ncas_RPS9B     69 TTTGAAATGAGAAAAGTAGAGAAGAA-----------------------TCACAGACCTACGCATGATTAAAGCGGACATTTAAAACACATTAT-------TTCAAATTG
Sbay_RPS9A    108 TTTGAAAGAGCTTTCGTTGAAATTAA----------------ATCATTTTCAAATAATGAA---CGATTCTT-----CGCCTGATACTTTTATT-------TTGGAAATA
Sbay_RPS9B     96 TATGAGAATATGAAATTAAAACACAC------------ATTCAAAACCTCCACCGAC--------------------AGACTGAAATACTTTCT-------ACCTTAACT
Scer_RPS9B     92 TTTGAGAATATGAAATTCCATAATAG----------------TATACCTTCATTGAGTAGC---ACGACAAC-----AGCCTGAATTACTATCC-------ATATTATGA
```

```
Klac_RPS9   226 --------------------------------TGTCGTTGAAAACCCGACACTTCAGCTCAAGGATAGAAACATTTTAATGCAAATCAATAAGAGCGAGAGCTCTTATTT
Scer_RPS9A  186 AAAACCT-------------------------TTACACTGAACTCCCGACACTTCAGGT--------AAAGCGTTCCGAAGTAATTCAACATTAGAAGAGGAACATTTCC
Egos_RPS9   108 ----------------------------------GCACTGGAAACCCGACACTCCAGTG---------------------------------------------------
Kthe_RPS9   149 ACACCTTGCTGGATACGCTT------------TTACTCTGAAAACCCGACACTTCAGGT--------AAAAGTACCA--GCTACACTGGTGCCTGCCGAGCTTGCTCTT-
Lwal_RPS9   161 ACATATCTC-----------TGGCCTTACTTTTTGCACTGAACACCCGACACTTCATGC--------AAATAGTGCCAGTATTTCATTAGGACTGTCTG------TTTCA
Cgla_RPS9A  267 CTGTACACA-----------------------GTACGCTAAATTCCCGACACTTTAGTC--------GTACTATCAT--CTT--AATAGCATGGGCCTA------TTTTT
Cgla_RPS9B  265 ATCGGAATGTAAGAAGTATCTTCTCTTTGGTTTGGCACTGAATTCCCGACACTTCAGGT--------CAAACATT-------------ATAACTACAGTATCTGTTTTTG
Vpol_RPS9A  198 ATCTTTA-------------------------TTGAATTGAACTCCCGACACTACAATT---------------------------------------------------
Vpol_RPS9B  181 CTATATGCAT----------------------TTGCACTGAACTCCCGACACTACAGGT--------AAATGTTGTA---------------------ATTTCACTTTCT
Zrou_RPS9   107 AAAGAT--------------------------TTCGTTTGAAAACCCGACACTTCAAAC--------GAAATTGTCC---------------------------------
Lklu_RPS9   175 TTGTACC-------------GTGGAATTATTTCTGCACTGAACTCCCGACACTTCAGGC--------AGAAACTCCA---------------------------------
Ncas_RPS9A  152 TC------------------------------TTTTACTGAATTCCCGACACTTCAGAT--------AAAGATATTC--TAGAGCTGACTTTATGTCAAATTGTATTTTT
Ncas_RPS9B  149 AATAGTTTTG----------------------TTTCACTGAATCCCCGACACTTCAGTAAAT-----GAAACTTTTC--GTTCTATGAATTTGAAATGTGTTGACTTTTC
Sbay_RPS9A  187 TAATCT--------------------------TTGCACTGAATTCCCGACACTTCAGGT-------AAAGTTTTTCCAAATCCTTCCAACATTTGTAGCATTCACCCCCT
Sbay_RPS9B  167 ACATCTTTAT----------------------TTACACTGAACTCCCGACACTTCAGTTAAA----AGAAAGATGTAGCAAACAATTAAGGTGATTCATGCGGACTTTCC
Scer_RPS9B  171 ATATCTTTAT----------------------TTACACTGAACTCCCGACACTTCAGTT--------AAACA--------------------------------------
                              Conserved stem-loop 4 ((((((..[[[[[..))))))

Klac_RPS9   304 AGTAC----TGTATGAATTAACACCTCATTCTGTTGTCAATTGACCCACAGAATGGACTTCGTTTTTTTCAGTTATACTTCTTTTTTGTTCATGTGTTTTCATGTTCTTA
Scer_RPS9A  263 CCAGT------------------TCTGATTATGTCTGTTGAAGATAACTCAAGTATTCTAGGCTTAA-------------------------------------------
Egos_RPS9   133 --------------------------------------------------------------------------------------------------------------
Kthe_RPS9   236 -----------------------------------CAGGCCCGGTTATTGTCATCTGGACTGCAATT-------------------------------------------
Lwal_RPS9   246 CAGGCACTTCTGTTGAAATTTGAAAGTCAAAGCCGAAAGGTTGCGTTTTCAAGTCGTGCTTATTGTG-------------------------------------------
Cgla_RPS9A  336 CTTCT----CGGGAGAATTGAGGTTCTTTTAGCTTCCAAGATGACTATGGTAGTGTAATCTGCCTCTGTATGTTACCACTTTTGGTTATGTACATAGTACTGAATGTTCA
Cgla_RPS9B  354 CTACTGCAGTGAGAGGTTTTGAGGATTTCTTAATACAGGATGTGTTAATGTCACTAGGTTGCTTTAG--------CTATTCCGTTTTAAGATAGAAAGTTAATGAACGA
Vpol_RPS9A  232 --------------------------CAATTATTTTAAGAAATATTTATGTCACACTCCCCCCTGTC-------------------------------------------
Vpol_RPS9B  240 CTAGT--------------------TTATTAAATATGAGCTAGAGACGTATCATGTTGAAAATTTTT-------------------------------------------
Zrou_RPS9   150 -------------AGTAATTGTGATACTGTTACTTTAA---TGGTTATCATGATGTCTTGTGCTAAT-------------------------------------------
Lklu_RPS9   231 ------CAGCTTTAATCATGAGACTTCTTGTTTTGTGATGCTGCTCAAAAGAAACTTTCTTTTTTTT-------------------------------------------
Ncas_RPS9A  222 ATCAT-----TTTAGTGATTACATACTCATTTTGTTCATATCTTTTGACAATTTGTAATAAACCAAT-------------------------------------------
Ncas_RPS9B  230 CCGTT---------------------TTTCAAAGTTGAGGCTGGATTTTCAAGCATTTTTCTTCTTT-------------------------------------------
Sbay_RPS9A  264 TCTAG---------GGGGTTGTCCTTTTACATTGTTGGTTGGAAGTATTTGAGCTTGATACAATTAT-------------------------------------------
Sbay_RPS9B  251 AAAGA----------------------CGTCTCTTTGGAGAGAATGAAAAGAGTATTTCATCCCTGT-------------------------------------------
Scer_RPS9B  213 -------------------GGGATACATTAGAGATCAAGGTGATCTAATAGGGAACATCTCTCTCGT-------------------------------------------

Klac_RPS9   410 AATGTTTGATGTGTCAATAGTTTAATGTTTCATGAGTAGATATTATATCGC------CCGTTTGTATATCCTCGT-----------------------------------
Scer_RPS9A  312 ---------TACAGAAATGTGTTAGGTTTGAGAACTCCATCATGGA-----------TTATGCATATTCGTCAGT---------------GACGTGCACAATTCTTTTGA
Egos_RPS9   133 ------------------------------------------------------------TGCGCAGGCCTCGGC-----------------------------------
Kthe_RPS9   268 ----------CAGTCAGAATCCAGATACAGTCGCGCTTGTTAT--------------GGACCTCTAGCCCTGACT-----------------------------------
Lwal_RPS9   313 ---------GGTATCTGCATTTTATATG-----------------------------CAGAGCCTACATGCAGGA-----------------------------------
Cgla_RPS9A  442 CTTGAAAATTTAGAATCATAGTGTAAAGGTGATACTAGCTGTTTTGCGATT------TGTAATTTGTTTCCTAGTATATGGTATTTAATCGTCTTAGTCTTTCATGGGTG
Cgla_RPS9B  456 GTTAATCATTTGGTAAATATTTTATGCAGACATGATTCATTACACTGATG-------AAATGCCTGAACATTGTT-----------------------------------
Vpol_RPS9A  273 ---------------------------------------------------------TCAGGCATAGAACACGTT-----------------------------------
Vpol_RPS9B  287 -------------GATATATACCTATTGAATGGAATGTACAATTCTGA---------TCTAACTTATTTATAAGT-----------------------------------
Zrou_RPS9   201 ---------------AGCATGTTGATT-----------------------------------------------------------------------------------
Lklu_RPS9   292 --TGGCAGCAGCTACAGCGTGCTATTCAATTTGTGCTGGCTCCTCTATTGCCAGGTTGAAGATTTCACTTTCAAT-----------------------------------
Ncas_RPS9A  284 ---------GGGGACGGTGTCCCAATTGGCCTAAACTTATT----------------GAATGTGAAGATCTTGAA-----------------------------------
Ncas_RPS9B  276 --------------------------------------------------------------TCTAGACTCCATT-----------------------------------
Sbay_RPS9A  322 -TTGTATCAGGTAGAAGAACTCTGTTCATGTACTATGGACGAACGC-----------CTCACCGTGTTAACCATT-----------------------------------
Sbay_RPS9B  296 ------------AGGCGGATACTGAATTAT---------------------------GCGTTCTTAACCACCATA-----------------------------------
Scer_RPS9B  261 ----AACAATGGGACAGTATTTTATTTTC----------------------------CAAGGCGGATACCTAATT-----------------------------------
```

```
Klac_RPS9      479 -------AGTGCAGTGATCTTTGGATTTTAGTATCAGATATATTGGGGACAACGAGTACATATGTGC-------------------------------------------
Scer_RPS9A     387 AGATAGGAAGAACAAAACTTCAATAATTGCTTTAATAATAT--------TTATGTAGTGA--------------------------------------------------
Egos_RPS9      148 -------------------------------------------------CAGTGGGCGGCG-------------------------------------------------
Kthe_RPS9      319 --------AGGGTCCAGTCTTTGAGATTGTCAGAACAATAT--------TCATGAGTAAACTGTA---------------------------------------------
Lwal_RPS9      350 ----------------------GATGGTATCGAAAAAGTAT--------TCATGAGTAGATTGTA---------------------------------------------
Cgla_RPS9A     547 TAGAAATCGTTTTGGATCCGTAGTATATGAATTCATGATATATGG----TTATAAATCCATAGCAAGTTGAAATTCATCACTTCTGTAGTCATCGTTGAAGCTATTTCTT
Cgla_RPS9B     524 -----------TACTTGGTGTAAGGGTTGATGAAAAAATTTTTGA----TCATGAACAGTAATATCA-------------------------------------------
Vpol_RPS9A     291 ---------------TCAATTAAGAATCTCCTTTGAATTAT--------TTATGATTGTATGGACC--------------------------------------------
Vpol_RPS9B     340 ------------GATAAGTGTTGCACGTCCCTTTTAAATTTCAATAGTACGAAGTTGACCTATCTCTTAAAGTTGAATGTCTGT--------------------------
Zrou_RPS9      213 ---------------------------TGATTCTCTAGTGT--------TTATGTGTGCACC------------------------------------------------
Lklu_RPS9      365 ---------TTATTGATGCTCTTTACTCAGCTCCAGTATAT--------TCATGAGTACATAAAC---------------------------------------------
Ncas_RPS9A     334 ------TGGAGCAAATGCCCTTGTTCTCGTCAACTTATTAT--------TTATGCACTGACCCA----------------------------------------------
Ncas_RPS9B     289 ------------------------------------AATATTTT-----ATATGAGTACCT-------------------------------------------------
Sbay_RPS9A     385 ----GATGAAGAATGATATTCAACAAGCGCCTTTATAATAT--------TTATGTACTGA--------------------------------------------------
Sbay_RPS9B     332 -----------------------------TCTCTGTTATAT--------TTATGAGCACTT-------------------------------------------------
Scer_RPS9B     304 ------------ATGCGTTTTTAATCATATCTCTACAATAT--------TTATGAGCACTTA------------------------------------------------

Klac_RPS9      539 -----ACGTGAGTTTTA--------------------------------CAATTATTTTGAATA----------------------------------------------
Scer_RPS9A     439 -----TCTTGATCCTCG--------------------------------CATTCGTTTGAAAAAGAGAG-----------------------------------------
Egos_RPS9      160 -----GCGCGAAGGACG--------------------------------GGACCGACTGGACGCGGC-------------------------------------------
Kthe_RPS9      368 -----GCCCGATCTTCG--------------------------------TAGCCTGTTC---------------------------------------------------
Lwal_RPS9      385 -----GCCCGTTCCTCG--------------------------------TAGTTATTGTTCATCTGAAC-----------------------------------------
Cgla_RPS9A     653 GGAGATTTCGAACCTCAATCACGACACACATATTTTACTGTGCAGTTATTACTCATTTTCCT---------------GAGATATATTTCCATGGATCA------------
Cgla_RPS9B     576 -----TCTGTAGTCTAGTTAATGGAGACCCG------------------TAGAACTTCTTATTTAGGAATTGTTAGCAGAAAAAATTGTCTGAACTAT------------
Vpol_RPS9A     334 -----TCTTGAACCTCTTTGCAGCA------------------------TAACTTCATTCTTGTATATGTATATACCATCACCACTCCCCTCCACAACTCAAGTGTTCAT
Vpol_RPS9B     412 -----TTTGGACCTTTG--------------------------------TAACCTCTTGATATCAATTAGCCAAATAGTGTAATTTAATGC-------------------
Zrou_RPS9      240 -----TCTTGAACCTCG--------------------------------TAAAACAATTGAATA----------------------------------------------
Lklu_RPS9      413 -----CCGTAAACCTCG--------------------------------TAGCATTAAGAATAT----------------------------------------------
Ncas_RPS9A     384 -----TCTTGAACCTCG--------------------------------TAATTTTTTCTTTCAGTTAAGAATTA-----------------------------------
Ncas_RPS9B     309 -----TCTTGAACCTCGTAGAAATCTCCATTGTTTTTTTA---------TTGCCATTTGAAACAAG--------------------------------------------
Sbay_RPS9A     433 -----TCTTGATCCTCA--------------------------------TTGCCCTTTATGCAAAAAGAGAG--------------------------------------
Sbay_RPS9B     356 -----TCTTGAACCTCT--------------------------------TGCACTATTTTTTACAAAGAAAC--------------------------------------
Scer_RPS9B     346 -----CTTGGGCCCTTG--------------------------------CAGACTTTTGTTCCGGGAAAAC---------------------------------------

Klac_RPS9      566 -TTTTCACTAACAAGAAATTA-----------------------------------------------------------------------------------------
Scer_RPS9A     471 -CGCTCACTAACAA-------------------------------------------------TGGTTGTAATTAT----------------------------------
Egos_RPS9      190 -CAGTAACTAACT-------------------------------------------------------------------------------------------------
Kthe_RPS9      390 -TTAAAACTAACA-------------------------------------------------------------------------------------------------
Lwal_RPS9      417 -CCATCACTAACG-------------------------------------------------------------------------------------------------
Cgla_RPS9A     736 -AGTTTACTAACAGTTAATGCCTTTTCGAAAATAAAGC-------TATGTTTTATGAGGTACATTGGTTGATTTGTAACGTTTGTTGCGGATACGATCTACACTACACAT
Cgla_RPS9B     651 -TTTTGACTAACACTTTATTTATACTTGTATGATGTGAGATTGTGTCTATTTGTTGAAGTGTTTTCTCTCACTTTTATCCTGAGCGTGATGCCGAATCAATAGCGATCTA
Vpol_RPS9A     415 TTTTAAACTAACAACATCAACTTTT--------------------------------------CCATTTAATTT------------------------------------
Vpol_RPS9B     466 -ATTTGACTAACA--------------------------------------------------TAAATTAATCATCACA-------------------------------
Zrou_RPS9      267 -TGTTAACTAAC--------------------------------------------------------------------------------------------------
Lklu_RPS9      440 -TTGTATCTAACAA------------------------------------------------------------------------------------------------
Ncas_RPS9A     422 -TTTAAACTAACA--------------------------------------------------TCATTT-----------------------------------------
Ncas_RPS9B     361 -CTGGGACTAACAATTTAAT-------------------------------------------TTTTTTAATTTTT----------------------------------
Sbay_RPS9A     468 -CACTCACTAACAAAA-----------------------------------------------TGATTTAATTAT-----------------------------------
Sbay_RPS9B     391 -TTTTGACTAACAAGT-----------------------------------------------TTATTTCATTATCTGTTTTT---------------------------
Scer_RPS9B     380 -TTTTGACTAACAAGAATC--------------------------------------------CAATTTTACTT------------------------------------
                         branchsite
```

```
Klac_RPS9     586 -------------------TTATC-------------CAAACAGGAGCCCCAAGAACTTACTCCAAGACTTACTCTACTCCAAAGAGACCTTACGAATCCGCTCGTTTGG
Scer_RPS9A    497 -------------------CTTGT-------------AAAACAGGAGCCCCAAGAACATATTCCAAGACTTACTCTACCCCAAAGAGACCTTACGAATCTTCTCGTTTGG
Egos_RPS9     202 -------------------------------------CGAGCAGGAGCCCCAAGAACCTACTCCAAGACGTACTCCACGCCAAAGAGACCATACGAGTCTGCGCGTCTAG
Kthe_RPS9     402 -------------------TCCTT----------------TCAGGAGCCCCAAGAACCTACTCTAAGACTTACTCTACCCCAAAGAGACCTTACGAGTCCGCTCGTCTGG
Lwal_RPS9     429 -------------------TTCCC-------------CCTTCAGGAGCCCCAAGAACCTACTCCAAGACTTACTCTACCCCAAAGAGACCTTACGAGTCTGCTCGTTTGG
Cgla_RPS9A    838 TTAGCATAAAAGAAGTTAATTTTTGAAAACTGAAATAAATACAGGAGCCCCAAGAACTTACTCTAAGACCTACTCTACCCCAAAGAGACCTTACGAATCTTCTCGTTTGG
Cgla_RPS9B    760 ATCACAAGTCTTTAAATTATTTTT----------------ATAGGAGCTCCAAGAACTTATTCTAAGACTTATTCTACCCCAAAGAGACCTTACGAATCTTCTCGTTTGG
Vpol_RPS9A    451 -------------------------------------AATTCAGGAGCCCCAAGAACTTACTCCAAGACCTACTCTACCCCAAAGAGACCATACGAATCTTCTCGTTTGG
Vpol_RPS9B    494 -------------------CTTTT-------------ATAACAGGAGCCCCAAGAACTTACTCTAAGACTTACTCTACCCCAAAGAGACCATACGAATCTTCTCGTTTGG
Zrou_RPS9     278 -------------------GTAGT-------------ACTTTAGGAGCCCCAAGAACCTACTCTAAGACCTACTCTACTCCAAAGAGACCATTCGAGTCTTCTCGTTTGG
Lklu_RPS9     453 -------------------TTTTT----------------ACAGGAGCTCCAAGAACTTACTCCAAGACTTACTCTACTCCAAAGAGACCATACGAATCTGCTCGTTTGG
Ncas_RPS9A    440 -------------------CTTTTAA-----------ACTATAGGAGCTCCAAGAACTTACTCAAAGACCTACTCAACTCCATCTAGACCATACGAATCTTCCCGTTTGG
Ncas_RPS9B    393 -------------------TTTTT-------------TAAATAGGAGCCCCAAGAACTTACTCTAAGACTTACTCCACTCCATCCAGACCATACGAATCTTCTCGTTTGG
Sbay_RPS9A    495 -------------------CTTGT-------------AAAACAGGAGCCCCAAGAACATACTCTAAGACCTATTCTACACCAAAGAGACCTTACGAATCTTCTCGTTTGG
Sbay_RPS9B    426 -------------------ATTTT-------------TCTATAGGAGCCCCAAGAACATACTCTAAGACTTACTCTACCCCAAAGAGACCTTACGAATCTTCTCGTTTGG
Scer_RPS9B    409 -------------------TTTTT-------------TTTTTAGGAGCTCCAAGAACTTACTCTAAGACTTACTCTACACCAAAGAGACCTTACGAATCTTCTCGTTTGG
                                                        3′ ss

Klac_RPS9     664 ACGCCGAATTGAAGTTGGCTGGTGAATACGGTTTGAAGAACAAGAGAGAAATTTACAGAATTTCTTTCCAATTGTCTAAGATCAGAAGAGCCGCCAGAGATTTGTTGACC
Scer_RPS9A    575 ACGCAGAATTGAAGTTGGCCGGTGAATTCGGTTTGAAGAACAAGAAGGAAATTTACAGAATTTCCTTCCAGTTATCTAAGATTCGTCGTGCTGCAAGAGACTTGTTGACA
Egos_RPS9     275 ACGGGAGTTGAAGCTAGCGGTGAGTACGGTTTGAAGAACAAGCGTGAAATCTACCGTATCTCGTTCCAGCTGTCCAAGATCAGACGTGCTGCCAGAGACTTGTTGACG
Kthe_RPS9     477 ACGCTGAGTTGAAGTTGGCTGGTGAATACGGCCTGAAGAACAAGAAGGAGATTTACAGAATCTCTTTCCAGTTGTCTAAGATCCGTCGTGCTGCCAGAGACTTGCTGACC
Lwal_RPS9     507 ACGCCGAGTTGAAGTTGGCCGGTGAATACGGTTTGAAGAACAAGAGAGAGATTTACAGAATCTCTTTCCAATTGTCTAAGATCCGTCGTGCTGCCAGAGACTTGCTGACC
Cgla_RPS9A    948 ACGCTGAATTGAAGCTTGCTGGTGAATTCGGTTTGAAGAACAAGAGAGAAATTTACAGAATTTCTTTCCAATTGTCCAAGATCAGAAGAGCTGCTAGAGATCTTTTGACC
Cgla_RPS9B    854 ACGCTGAATTGAAGCTTGCTGGTGAATTCGGTTTGAAGAACAAGAGAGAAATTTACAGAATTTCTTTCCAATTGTCTAAGATCAGAAGAGCTGCTAGAGATCTTTTGACC
Vpol_RPS9A    524 ACGCTGAATTGAAATTGGCTGGTGAATTCGGTTTGAAGAACAAGAGAGAAATCTACAGAATTTCTTTCCAATTGTCTAAGATTCGTCGTGCTGCCAGAGACTTATTGACC
Vpol_RPS9B    572 ACGCTGAATTGAAATTGGCTGGTGAATTCGGTTTGAAGAACAAGAGAGAAATCTACAGAATTTCTTTCCAATTGTCTAAGATTCGTCGTGCTGCCAGAGACTTATTGACC
Zrou_RPS9     356 ACGCCGAATTGAAAATTGCCGGTGAATATGGTTTGAGAAACAAGAGAGAAATTTACAGAATTACTTTCCAATTGTCTAAGATTCGTCGTGCTGCCAGAGAATTGTTGACC
Lklu_RPS9     528 ACGCTGAATTGAAGTTGGCCGGTGAATACGGTTTGAAGAACAAGAGAGAGATTTACAGAATTTCTTTCCAATTGTCTAAGATTCGTCGTGCTGCCAGAGACTTGTTGACC
Ncas_RPS9A    520 ACGCCGAATTGAAATTGGCTGGTGAATTCGGTTTGAAAAACAAGAGAGAAATTTACAGAATTTCTTTCCAATTATCTAAGATTCGTCGTGCCGCTAGAGACTTGTTAACT
Ncas_RPS9B    471 ACGCTGAATTGAAGTTGGCCGGTGAATTCGGTTTGAAGAACAAGAGAGAAATTTACAGAATTTCTTTCCAATTATCCAAGATTCGTCGTGCTGCCAGAGATTTATTGACC
Sbay_RPS9A    573 ATGCTGAATTGAAGTTGGCCGGTGAATTCGGTTTGAAGAACAAGAAGGAAATTTACAGAATTTCTTTCCAATTGTCCAAGATTCGTCGTGCTGCCAGAGATTTATTGACC
Sbay_RPS9B    504 ACGCTGAATTGAAGTTGGCCGGTGAATTCGGTTTGAAGAACAAGAAGGAAATTTACAGAATTTCTTTCCAATTGTCTAAGATTCGTCGTGCTGCCAGAGATTTATTGACC
Scer_RPS9B    487 ACGCAGAATTGAAGTTGGCCGGTGAATTCGGTTTGAAGAACAAGAGAGAAATTTACAGAATTTCTTTCCAATTGTCTAAAATTCGTCGTGCTGCCAGAGACTTGTTAACT

Klac_RPS9     774 AGAGACGAAAAGGACCCAAAGAGATTGTTCGAAGGTAATGCTTTGATCAGAAGATTGGTGAGAATTGGTGTCTTGTCTGAAGACAAGAAGAAGTTGGATTATGTCTTGGC
Scer_RPS9A    685 AGAGACGAAAAGGACCCAAAGAGATTGTTCGAAGGTAATGCTTTGATTAGGAGACTAGTTAGAGTCGGTGTTTTATCCGAAGATAAGAAGAAGTTAGATTATGTTTTAGC
Egos_RPS9     385 CGTGACGAGAAGGACCCTAAGCGTCTGTTCGAGGGTAACGCGTTGATCCGCCGTCTTGTGAGAATCGGTGTCTTGTCCGAGGACAAGAAGAAGTTGGATTACGTGTTGGC
Kthe_RPS9     587 AGAGATGAGAAGGACCCAAGAAGACTTTTCGAGGGTAACGCCATGATCAGAAGACTGGTCAGACTTGGTGTCTTGTCCGAGGACAAGAAGAAGTTGGATCACGTCTTGGC
Lwal_RPS9     617 AGAGATGAGAAGGACCCAAGAAGACTTTTCGAGGGTAACGCCATGATCAGAAGATTGGTGAGACTAGGTGTCTTGTCTGAGGACAAGAAGAAGCTGGATTACGTCTTGGC
Cgla_RPS9A   1058 AGAGACGAAAAGGACCCAAAGAGATTGTTCGAAGGTAATGCTTTGATCAGAAGATTGGTGAGAATCGGTGTCTTGTCCGAAGACAAGAAGAAGTTGGATTATGTCTTGGC
Cgla_RPS9B    964 AGAGACGAAAAGGACCCAAAGAGATTGTTCGAAGGTAATGCTTTGATCAGAAGATTGGTGAGAATCGGTGTCTTGTCCGAAGACAAGAAGAAGTTGGATTATGTCTTGGC
Vpol_RPS9A    634 AGAGACGAAAAAGACCCAAAGAGATTATTCGAAGGTAATGCTTTGATCAGAAGATTGACCAGAGTCGGTGTCTTATCTGAAGACAAGAGAAAGTTAGATTATGTCTTGGC
Vpol_RPS9B    682 AGAGACGAAAAAGACCCAAAGAGATTATTCGAAGGTAATGCTTTGATCAGAAGATTGACCAGAGTCGGTGTCTTATCTGAAGACAAGAGAAAATTAGATTATGTCTTGGC
Zrou_RPS9     466 AAGGACGAAAAAGACCAAAAGAGATTATTCGAAGGTAATGCCTTGATCAGAAGATTGGTCAGAGTGGGTGTCTTGTCTGAAGACAAAAAGAAGCTCGATTATGTCTTGGC
Lklu_RPS9     638 AGAGATGAAAAGGACCCAAAGAGATTGTTCGATGGTAACGCCTTGATCAGAAGATTGGTGAGAATCGGTGTCTTGTCCGAAGACAAGAAGAAGTTGGATTACGTCTTGGC
Ncas_RPS9A    630 AGAGATGAAAAGGATCCAAAGAGATTATTCGAAGGTAATGCCTTGATCAGAAGATTAGTGAGAATTGGTGTCTTGTCTGAAGATAAGAAGAAGTTAGATTATGTTTTAGC
Ncas_RPS9B    581 AGAGATGAAAAGGACCCAAAGAGATTGTTCGAAGGTAATGCCTTGATCAGAAGATTGGTGAGAATTGGTGTCTTGTCCGAAGATAAGAAGAAGTTAGATTATGTTTTGGC
Sbay_RPS9A    683 AGAGACGAAAAGGACCCAAAGAGATTGTTCGAAGGTAATGCTTTGATCAGAAGATTGGTTAGAATCGGTGTCTTGTCCGAAGACAAGAAGAAGTTGGATTATGTCTTAGC
Sbay_RPS9B    614 AGAGACGAAAAGGACCCAAAGAGATTGTTCGAAGGTAACGCTTTGATCAGAAGATTGGTTAGAATTGGTGTCTTGTCCGAAGACAAGAAGAAGTTGGATTATGTTTTAGC
Scer_RPS9B    597 AGAGACGAAAAGGACCCAAAGAGATTGTTCGAAGGTAATGCCTTGATCAGAAGATTGGTTAGAGTTGGTGTCTTGTCCGAAGACAAGAAGAAGTTGGATTATGTTTTGGC
```

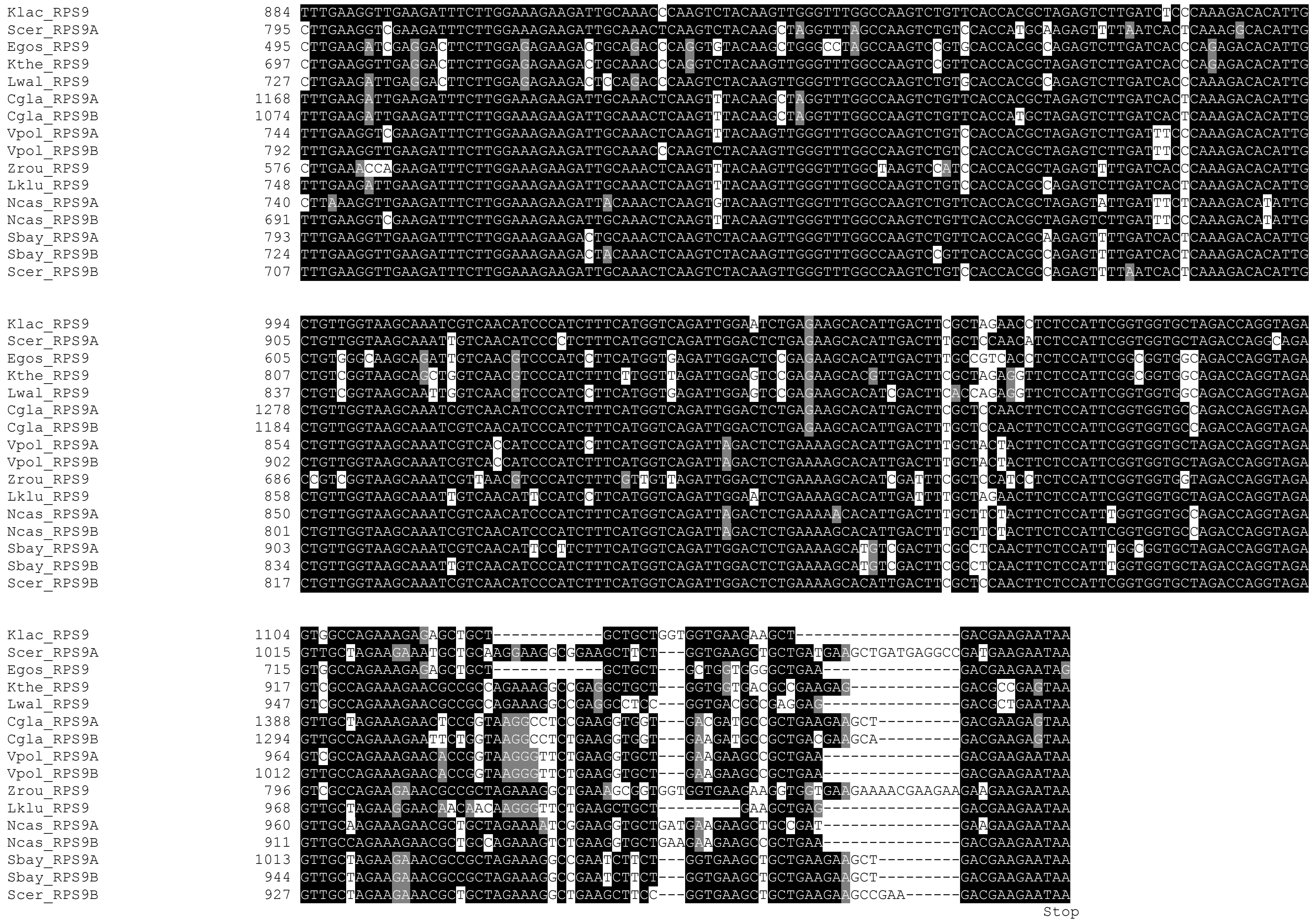
Klac_RPS9 884
Scer_RPS9A 795
Egos_RPS9 495
Kthe_RPS9 697
Lwal_RPS9 727
Cgla_RPS9A 1168
Cgla_RPS9B 1074
Vpol_RPS9A 744
Vpol_RPS9B 792
Zrou_RPS9 576
Lklu_RPS9 748
Ncas_RPS9A 740
Ncas_RPS9B 691
Sbay_RPS9A 793
Sbay_RPS9B 724
Scer_RPS9B 707
Klac_RPS9 994
Scer_RPS9A 905
Egos_RPS9 605
Kthe_RPS9 807
Lwal_RPS9 837
Cgla_RPS9A 1278
Cgla_RPS9B 1184
Vpol_RPS9A 854
Vpol_RPS9B 902
Zrou_RPS9 686
Lklu_RPS9 858
Ncas_RPS9A 850
Ncas_RPS9B 801
Sbay_RPS9A 903
Sbay_RPS9B 834
Scer_RPS9B 817
Klac_RPS9 1104
Scer_RPS9A 1015
Egos_RPS9 715
Kthe_RPS9 917
Lwal_RPS9 947
Cgla_RPS9A 1388
Cgla_RPS9B 1294
Vpol_RPS9A 964
Vpol_RPS9B 1012
Zrou_RPS9 796
Lklu_RPS9 968
Ncas_RPS9A 960
Ncas_RPS9B 911
Sbay_RPS9A 1013
Sbay_RPS9B 944
Scer_RPS9B 927
Stop
