# Supplementary figures and images for: Diverse Forms of RPS9 Splicing Are Part of an Evolving Autoregulatory Circuit
Source: PLoS Genet. 2012 Mar 29;8(3):e1002620. doi: 10.1371/journal.pgen.1002620 (PMC3315480; doi:10.1371/journal.pgen.1002620)

## RPS14

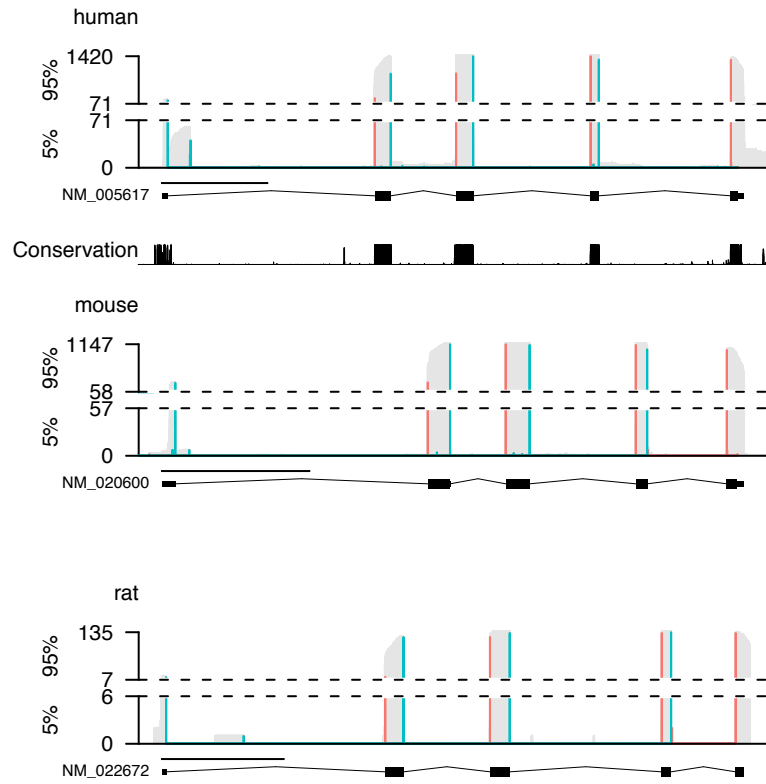

## RPL30

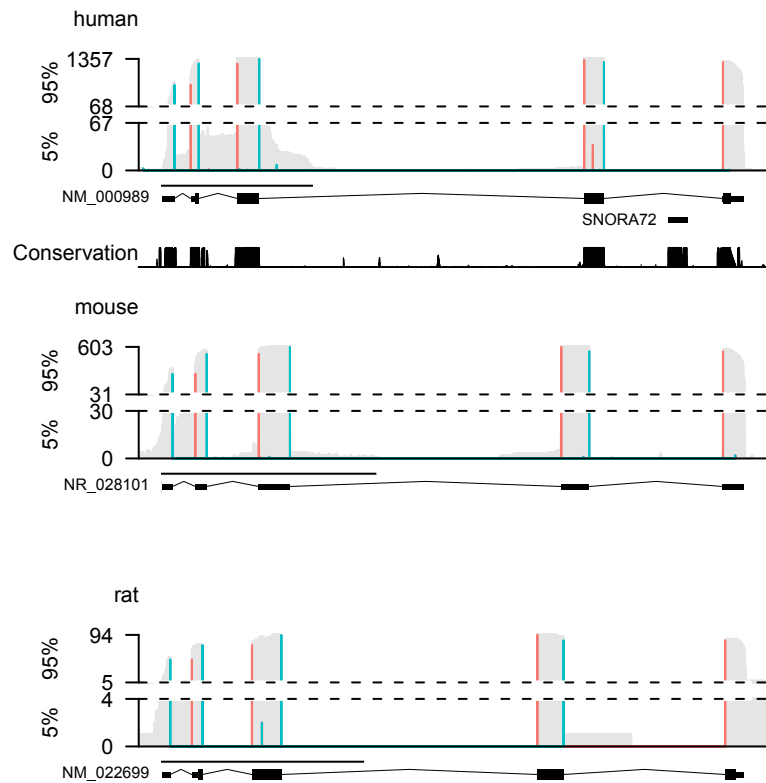

Supplement: Figure S3 — EST analysis of mammalian RPL30 and RPS14 does not reveal conserved alternatively-spliced isoforms. EST summaries of RPL30 and RPS14 orthologs from human, mouse, and rat illustrated as in Figure 4. Genes are plotted to scale (black line; 1 kb). (PDF) [file pgen.1002620.s003.pdf]

A

## Conserved RNA structures in the RPS9A gene

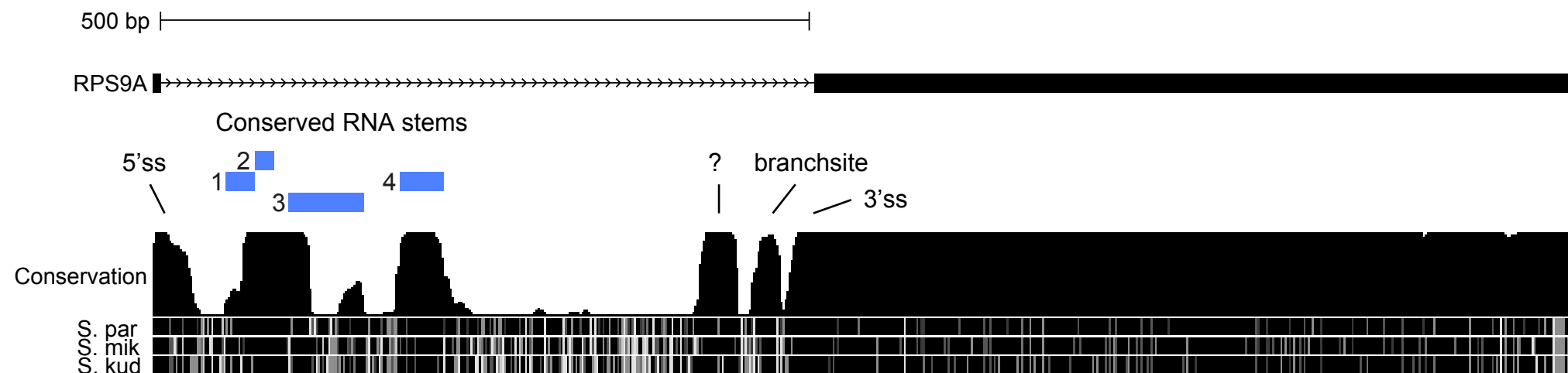

B

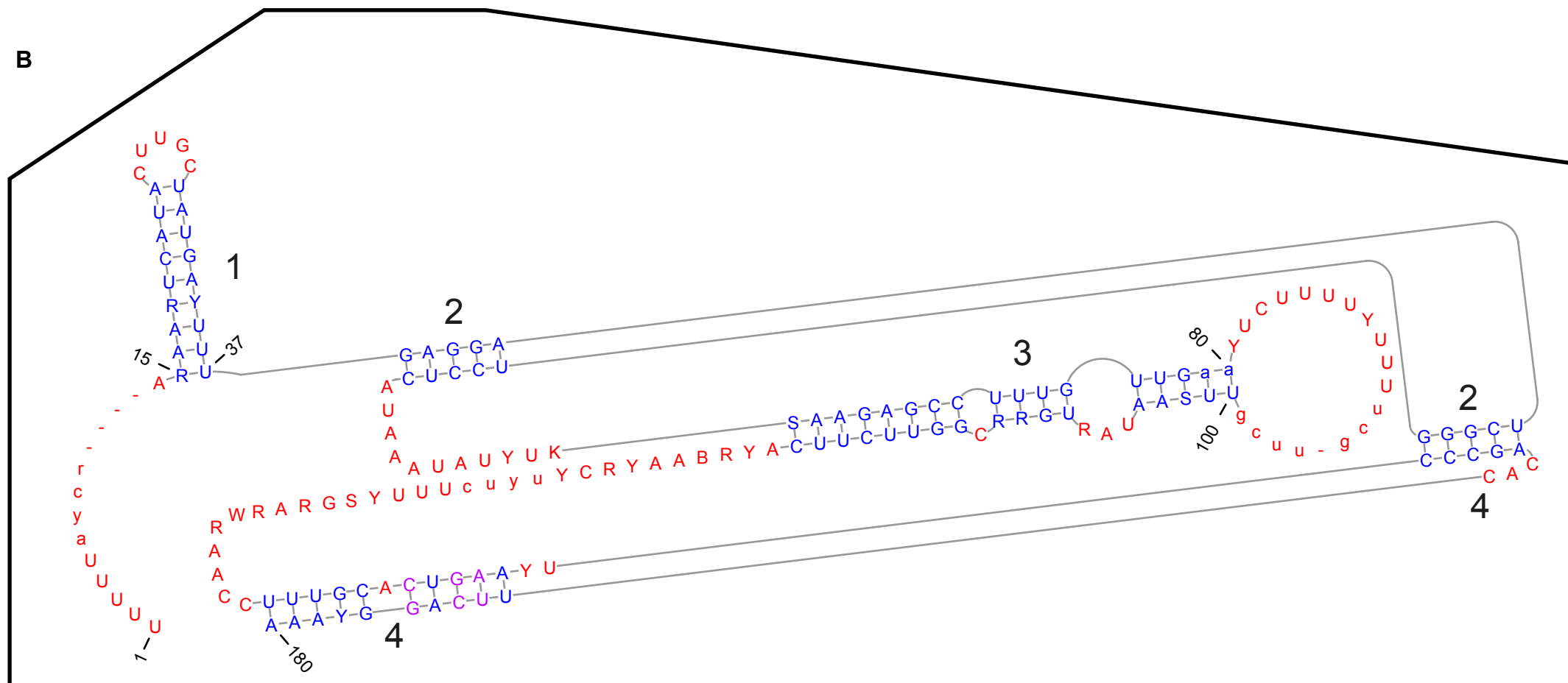

Supplement: Figure S4 — Putative RNA structure within the RPS9A and RPS9B introns. Conserved elements in the RPS9A and RPS9B introns are associated with a putative pseudoknot structure near the 5′ splice site. A) An illustration of the S. cerevisiae RPS9A gene model and nucleotide conservation among closely related yeasts (from the UCSC Genome Browser). Putative RNA stems (predicted by the RNAz program [26]) that overlap with conserved regions are indicated (numbered 1–4). B) Illustration of a putative H-H type pseudoknot (predicted by the IPknot program [64]) based on nucleotide alignment of pre- and post-WGD yeasts (Figure S2). Positions of compensatory base pair changes that support the pseudoknot stems are indicated in purple. Pseudoknot illustration was created with PseudoViewer v3.0 (http://pseudoviewer.inha.ac.kr/). (PDF) [file pgen.1002620.s004.pdf]
