# Supplementary material for: Diverse Forms of RPS9 Splicing Are Part of an Evolving Autoregulatory Circuit
Source: PLoS Genet. 2012 Mar 29;8(3):e1002620. doi: 10.1371/journal.pgen.1002620 (PMC3315480; doi:10.1371/journal.pgen.1002620)
Supplement: Table S1 — Intron annotations and predictions. (PDF) [file pgen.1002620.s005.pdf]

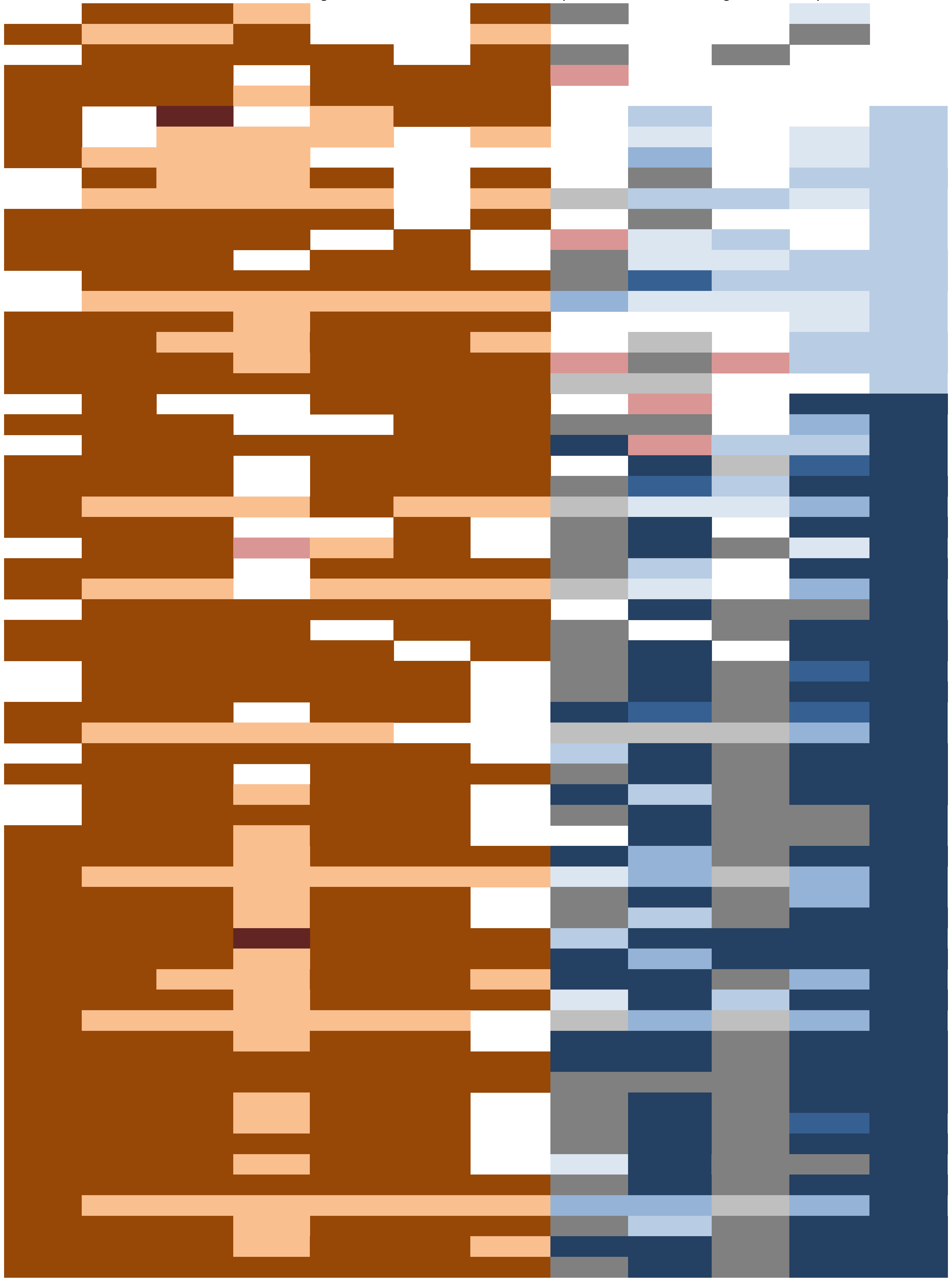

Gene.1	Gene.2	Name.1	Name.2	Gene class	Calb	Lklu	Lthe	Lwal	Egos	Klac	Zrou	Vpol	Ncas	Cgla	Sbay	Scer	preWGD Ancestor
Scer_YGOB_SDC25	YLR310C	NA	PUS1	non-RPG													1
Scer_YGOB_YDR134C	YLR110C	NA	FKS1	non-RPG													1
YAL015C	YOL043C	NTG1		non-RPG													1
YAL037W	YOR342C		SKI7	non-RPG													1
YAL051W	YOR363C	OAF1	YPT32	non-RPG													1
YAL056W	YOR371C	GPB2	HRB1	non-RPG													1
YAL062W	YOR375C	GDH3	NHP6A	non-RPG													1
YAR002C-A	YGL002W	ERP1	RIM11	non-RPG													1
YBL005W	YGL013C	PDR3	SEC14	non-RPG													1
YBL009W	YGL021W	ALK2	APE2	non-RPG													1
YBL039C	YJR103W	URA7	SCS2	non-RPG													1
YBL043W	YJR115W	ECM13	SRC1	non-RPG													1
YBL061C	YER096W	SKT5	PST1	non-RPG													1
YBL067C	YER098W	UBP13		non-RPG													1
YBL068W	YER099C	PRS4	ARF1	non-RPG													1
YBL069W	YER101C	AST1	SNC2	non-RPG													1
YBL079W	YER105C	NUP170		non-RPG													1
YBL101C	YPR030W	ECM21	IMD3	non-RPG													1
YBL106C	YPR032W	SRO77	COX5B	non-RPG													1
YBR001C	YDR001C	NTH2	CPT1	non-RPG													1
YBR014C	YDL010W	GRX7	UBC5	non-RPG													1
YBR068C	YDR046C	BAP2	AIM11	non-RPG													1
YBR104W	YPR058W	YMC2	TUB1	non-RPG													1
YBR117C	YPR074C	TKL2	YSC84	non-RPG													1
YBR145W	YOL086C	ADH5	SUN4	non-RPG													1
YBR150C	YOL089C	TBS1	RPL14A	RPG													1
YBR177C	YPL095C	EHT1	RPL17A	RPG													1
YBR183W	YPL087W	YPC1	RPL27A	RPG													1
YBR214W	YGL056C	SDS24	RPS26A	RPG													1
YBR270C	YJL058C	BIT2	RPL40B	RPG													1
YBR278W	YJL065C	DPB3	RPL43A	RPG													1
YBR284W	YJL070C		RPS10A	RPG													1
YCL036W	YDR514C	GFD2	RPL13B	RPG													1
YCL037C	YDR515W	SRO9	RPS24B	RPG													1
YCR024C-A	YEL017C-A	PMP1	RPL2B	RPG													1
YCR026C	YEL016C	NPP1	RPL24B	RPG													1
YCR048W	YNR019W	ARE1	RPS23B	RPG													1
YCR052W	YNR023W	RSC6	RPS27A	RPG													1
YCR073W-A	YNR034W	SOL2	RPL16B	RPG													1
YCR089W	YNR044W	FIG2	RPS7A	RPG													1
YDL019C	YAR042W	OSH2	RPS4A	RPG													1
YDL036C	YOL066C	PUS9	RPL19A	RPG													1
YDL042C	YOL068C	SIR2	RPS8B	RPG													1
YDL048C	YLR375W	STP4	RPL23B	RPG													1
YDL070W	YLR399C	BDF2	RPS11A	RPG													1
YDL085W	YMR145C	NDE2	RPS6A	RPG													1
YDL155W	YLR210W	CLB3	RPS9A	RPG													1
YDL179W	YDL127W	PCL9	RPL21B	RPG													1
YDL181W	YDL130W-A	INH1	RPS14B	RPG													1
YDL188C	YDL134C	PPH22	RPS29A	RPG													1
YDL194W	YDL138W	SNF3	RPL31B	RPG													1
YDL204W	YDR233C	RTN2	RPS16A	RPG													1
YDL222C	YNL194C	FMP45	RPL35B	RPG													1
YDL224C	YNL197C	WHI4	RPS17A	RPG													1
YDL226C	YNL204C	GCS1	RPS18B	RPG													1
YDL229W	YNL209W	SSB1	RPL37A	RPG													1
YDL239C	YNL225C	ADY3	RPL34B	RPG													1
YDR034W-B	YBR056W-A		RPL22A	RPG													1
YDR085C	YER158C	AFR1	RPS25B	RPG													1
YDR093W	YER166W	DNF2	RPL26A	RPG													1
YDR096W	YER169W	GIS1	RPS0B	RPG													1
YDR098C	YER174C	GRX3	RPL42A	RPG													1

Gene.1	Gene.2	Name.1	Name.2	Gene class	Calb	Lklu	Lthe	Lwal	Egos	Klac	Zrou	Vpol	Ncas	Cgla	Sbay	Scer	preWGD Ancestor
YDR107C	YLR083C	TMN2	RPS21A	RPG													1
YDR111C	YLR089C	ALT2	RPS30B	RPG													1
YDR125C	YLR099C	ECM18	RPL6A	RPG													1
YDR132C	YLR108C		RPL36B	RPG													1
YDR144C	YLR120C	MKC7	RPL20B	RPG													1
YDR151C	YLR136C	CTH1	RPL18A	RPG													1
YDR178W	YLR164W	SDH4	RPS19A	RPG													1
YDR206W	YLR233C	EBS1	RPL33A	RPG													1
YDR222W	YLR225C		CDC25	non-RPG													0
YDR223W	YLR223C	CRF1	CCW12	non-RPG													0
YDR251W	YPL032C	PAM1	NTG2	non-RPG													0
YDR259C	YOR028C	YAP6		non-RPG													0
YDR277C	YOR047C	MTH1	PIP2	non-RPG													0
YDR300C	YHR033W	PRO1	GPB1	non-RPG													0
YDR303C	YHR056C	RSC3	GDH1	non-RPG													0
YDR309C	YHR061C	GIC2	ERP6	non-RPG													0
YDR312W	YHR066W	SSF2	PDR1	non-RPG													0
YDR326C	YHR080C	YSP2	ALK1	non-RPG													0
YDR342C	YHR092C	HXT7	URA8	non-RPG													0
YDR351W	YHR103W	SBE2		non-RPG													0
YDR353W	YHR106W	TRR1	SHC1	non-RPG													0
YDR358W	YHR108W	GGA1	UBP9	non-RPG													0
YDR389W	YOR134W	SAC7	PRS2	non-RPG													0
YDR436W	YML016C	PPZ2	AST2	non-RPG													0
YDR438W	YML018C	THI74	NUP157	non-RPG													0
YDR463W	YHR006W	STP1	CSR2	non-RPG													0
YDR492W	YOL101C	IZH1	SRO7	non-RPG													0
YDR497C	YOL103W	ITR1	NTH1	non-RPG													0
YDR502C	YLR180W	SAM2	GRX6	non-RPG													0
YDR505C	YLR177W	PSP1	BAP3	non-RPG													0
YEL006W	YIL006W	YEA6	YMC1	non-RPG													0
YEL041W	YJR049C	YEF1	TKL1	non-RPG													0
YEL047C	YJR051W		ADH1	non-RPG													0
YEL060C	YOR003W	PRB1	HAL9	non-RPG													0
YER027C	YGL208W	GAL83	EEB1	non-RPG													0
YER028C	YGL209W	MIG3	YDC1	non-RPG													0
YER035W	YGL222C	EDC2	SDS23	non-RPG													0
YER039C	YGL225W	HVG1	BIT61	non-RPG													0
YER054C	YIL045W	GIP2	DLS1	non-RPG													0
YER057C	YIL051C	HMF1		non-RPG													0
YER062C	YIL053W	HOR2		non-RPG													0
YER067W	YIL057C	RGI1	SLF1	non-RPG													0
YER070W	YIL066C	RNR1	PMP2	non-RPG													0
YFL018C	YPL017C	LPD1	NPP2	non-RPG													0
YFL042C	YLR072W		ARE2	non-RPG													0
YFL048C	YLR080W	EMP47	SNF12	non-RPG													0
YFL060C	YNL334C	SNO3	SOL1	non-RPG													0
YFR012W	YOL019W		AGA1	non-RPG													0
YFR013W	YOL017W	IOC3	SWH1	non-RPG													0
YFR014C	YOL016C	CMK1	RIB2	non-RPG													0
YFR017C	YOL024W	IGD1	HST1	non-RPG													0
YFR023W	YHR015W	PES4	STP3	non-RPG													0
YFR039C	YGL228W		BDF1	non-RPG													0
YFR053C	YGL253W	HXK1	NDE1	non-RPG													0
YGL049C	YGR162W	TIF4632	CLB4	non-RPG													0
YGL082W	YPL191C		PCL2	non-RPG													0
YGL133W	YPL216W	ITC1	STF1	non-RPG													0
YGL134W	YPL219W	PCL10	PPH21	non-RPG													0
YGL139W	YPL221W	FLC3	RGT2	non-RPG													0
YGL162W	YPR009W	SUT1	RTN1	non-RPG													0
YGR010W	YLR328W	NMA2		non-RPG													0
YGR038W	YLR350W	ORM1	WHI3	non-RPG													0

Gene.1	Gene.2	Name.1	Name.2	Gene class	Calb	Lklu	Lthe	Lwal	Egos	Klac	Zrou	Vpol	Ncas	Cgla	Sbay	Scer	preWGD Ancestor
YGR041W	YLR353W	BUD9	SPS18	non-RPG													0
YGR043C	YLR354C	NQM1	SSB2	non-RPG													0
YGR049W	YLR356W	SCM4	CNM67	non-RPG													0
YGR056W	YLR357W	RSC1		non-RPG													0
YGR070W	YLR371W	ROM1		non-RPG													0
YGR071C	YLR373C		DNF1	non-RPG													0
YGR097W	YPR115W	ASK10	RPH1	non-RPG													0
YGR108W	YPR119W	CLB1	GRX4	non-RPG													0
YGR121C	YPR138C	MEP1	EMP70	non-RPG													0
YGR124W	YPR145W	ASN2	ALT1	non-RPG													0
YGR131W	YPR149W	FHN1	ICT1	non-RPG													0
YGR138C	YPR156C	TPO2		non-RPG													0
YGR141W	YPR157W	VPS62	YPS1	non-RPG													0
YGR143W	YPR159W	SKN1	TIS11	non-RPG													0
YGR169C-A	YJR005C-A			non-RPG													0
YGR209C	YLR043C	TRX2	EST1	non-RPG													0
YGR213C	YLR046C	RTA1		non-RPG													0
YGR238C	YHR158C	KEL2	IFH1	non-RPG													0
YGR241C	YHR161C	YAP1802	SVL3	non-RPG													0
YGR254W	YHR174W	ENO1	CIN5	non-RPG													0
YHR001W	YKR003W	OSH7	STD1	non-RPG													0
YHR030C	YKL161C	SLT2		non-RPG													0
YHR115C	YNL116W	DMA1	RSC30	non-RPG													0
YHR132W-A	YNL157W	IGO2	GIC1	non-RPG													0
YHR135C	YNL154C	YCK1	SSF1	non-RPG													0
YHR146W	YNL173C	CRP1		non-RPG													0
YHR155W	YNL257C	YSP1	HXT4	non-RPG													0
YHR206W	YJR147W	SKN7	SBE22	non-RPG													0
YIL079C	YDL175C	AIR1	TRR2	non-RPG													0
YIL109C	YNL049C	SEC24	GGA2	non-RPG													0
YIL113W	YNL053W	SDP1	BAG7	non-RPG													0
YIL117C	YNL058C	PRM5	PPZ1	non-RPG													0
YIL135C	YNL074C	VHS2		non-RPG													0
YIL149C	YKR095W	MLP2	STP2	non-RPG													0
YIL158W	YKR100C	AIM20	IZH4	non-RPG													0
YIR021W	YKL011C	MRS1	ITR2	non-RPG													0
YIR037W	YKL026C	HYR1	SAM1	non-RPG													0
YJL045W	YKL148C			non-RPG													0
YJL076W	YKR010C	NET1	YIA6	non-RPG													0
YJL079C	YKR013W	PRY1	UTR1	non-RPG													0
YJL082W	YKR018C	IML2	OSM1	non-RPG													0
YJL083W	YKR019C	TAX4	YSP3	non-RPG													0
YJL110C	YKR034W	GZF3	SIP2	non-RPG													0
YJL116C	YKR042W	NCA3	MIG2	non-RPG													0
YJL129C	YKR050W	TRK1	EDC1	non-RPG													0
YJL139C	YKR061W	YUR1	VRG4	non-RPG													0
YJL164C	YKL166C	TPK1	PIG2	non-RPG													0
YJL181W	YJR030C		MMF1	non-RPG													0
YJR059W	YKL198C	PTK2	RHR2	non-RPG													0
YKL043W	YMR016C	PHD1	RGI2	non-RPG													0
YKL065C	YMR040W	YET1	RNR3	non-RPG													0
YKL104C	YMR085W	GFA1	IRC15	non-RPG													0
YKL105C	YMR086W			non-RPG													0
YKL121W	YMR102C	DGR2	EMP46	non-RPG													0
YKL129C	YMR109W	MYO3	SNO2	non-RPG													0
YKL132C	YMR113W	RMA1		non-RPG													0
YKL133C	YMR115W		ESC8	non-RPG													0
YKL141W	YMR118C	SDH3	CMK2	non-RPG													0
YKL187C	YLR413W			non-RPG													0
YKR072C	YOR054C	SIS2	MIP6	non-RPG													0
YKR105C	YCL069W	VBA5	SHE10	non-RPG													0
YLL010C	YLR019W	PSR1	HXK2	non-RPG													0

Gene.1	Gene.2	Name.1	Name.2	Gene class	Calb	Lklu	Lthe	Lwal	Egos	Klac	Zrou	Vpol	Ncas	Cgla	Sbay	Scer	preWGD Ancestor
YLL021W	YLR313C	SPA2	TIF4631	non-RPG													0
YLL051C	YKL220C	FRE6		non-RPG													0
YLL061W	YPL274W	MMP1		non-RPG													0
YLR031W	YMR124W		PCL8	non-RPG													0
YLR174W	YNL009W	IDP2	FLC1	non-RPG													0
YLR249W	YNL014W	YEF3	SUT2	non-RPG													0
YLR260W	YOR171C	LCB5	NMA1	non-RPG													0
YLR266C	YOR162C	PDR8	ORM2	non-RPG													0
YLR273C	YOR178C	PIG1	BUD8	non-RPG													0
YLR284C	YOR180C	ECI1	TAL1	non-RPG													0
YLR433C	YML057W	CNA1	ATG33	non-RPG													0
YLR442C	YML065W	SIR3	RSC2	non-RPG													0
YLR450W	YML075C	HMG2	ROM2	non-RPG													0
YLR456W	YPR172W		VID22	non-RPG													0
YLR457C	YPR174C	NBP1	RGC1	non-RPG													0
YML106W	YMR271C	URA5	CLB2	non-RPG													0
YML111W	YMR275C	BUL2	MEP3	non-RPG													0
YML118W	YMR285C	NGL3	ASN1	non-RPG													0
YMR008C	YOL011W	PLB1	NCE102	non-RPG													0
YMR175W	YPL223C	SIP18	TPO3	non-RPG													0
YMR180C	YPL228W	CTL1	TDA6	non-RPG													0
YMR181C	YPL229W		KRE6	non-RPG													0
YMR183C	YPL232W	SSO2		non-RPG													0
YMR192W	YPL249C	GYL1	TRX1	non-RPG													0
YMR198W	YPL253C	CIK1		non-RPG													0
YMR199W	YPL256C	CLN1	KEL1	non-RPG													0
YMR251W-A	YOL052C-A	HOR7	YAP1801	non-RPG													0
YNL001W	YCL001W-B	DOM34	ENO2	non-RPG													0
YNL065W	YIL120W	AQR1	OSH6	non-RPG													0
YNL093W	YOR089C	YPT53	KDX1	non-RPG													0
YNL270C	YEL063C	ALP1	DMA2	non-RPG													0
YNL283C	YOL105C	WSC2	IGO1	non-RPG													0
YNL293W	YOL112W	MSB3	YCK2	non-RPG													0
YNL298W	YOL113W	CLA4	MDG1	non-RPG													0
YNL299W	YOL115W	TRF5	SIP3	non-RPG													0
YOL036W	YIR016W		HMS2	non-RPG													0
YOR016C	YAL007C	ERP4	AIR2	non-RPG													0
YOR153W	YDR406W	PDR5	SFB2	non-RPG													0
YOR186W	YLR297W		MSG5	non-RPG													0
YOR222W	YPL134C	ODC2		non-RPG													0
YOR226C	YPL135W	ISU2	MLF3	non-RPG													0
YOR231W	YPL140C	MKK1	MLP1	non-RPG													0
YOR247W	YPL163C	SRL1	SKG1	non-RPG													0
YOR381W	YNR060W	FRE3	CCE1	non-RPG													0
YPL112C	YOR193W	PEX25	GPX1	non-RPG													0
YPL130W	YOR214C	SPO19	SDH1	non-RPG													0
YPL250C	YMR195W	ICY2	TOF2	non-RPG													0
YPR162C	YLR453C	ORC4	PRY2	non-RPG													0
YAL017W	YOL045W	PSK1		non-RPG													0
YAL029C	YOR326W	MYO4	IRS4	non-RPG													0
YAL034C	YOR338W	FUN19	DAL80	non-RPG													0
YBL054W	YER088C	TOD6	UTH1	non-RPG													0
YBL075C	YER103W	SSA3	TRK2	non-RPG													0
YBR049C	YDR026C	REB1	KTR2	non-RPG													0
YBR054W	YDR033W	YRO2	TPK3	non-RPG													0
YBR118W	YPR080W	TEF2		non-RPG													0
YBR161W	YPL057C	CSH1	PTK1	non-RPG													0
YBR172C	YPL105C	SMY2	SOK2	non-RPG													0
YBR273C	YJL048C	UBX7	YET2	non-RPG													0
YCL024W	YDR507C	KCC4		non-RPG													0
YCL025C	YDR508C	AGP1		non-RPG													0
YCL035C	YDR513W	GRX1		non-RPG													0

Gene.1	Gene.2	Name.1	Name.2	Gene class	Calb	Lklu	Lthe	Lwal	Egos	Klac	Zrou	Vpol	Ncas	Cgla	Sbay	Scer	preWGD Ancestor
YCL040W	YDR516C	GLK1	MYO5	non-RPG													0
YCL043C	YDR518W	PDI1	FOL3	non-RPG													0
YCR067C	YNR026C	SED4	MGR3	non-RPG													0
YCR069W	YNR028W	CPR4		non-RPG													0
YCR094W	YNR048W	CDC50		non-RPG													0
YDL021W	YOL056W	GPM2	VHS3	non-RPG													0
YDL109C	YGL144C		VBA3	non-RPG													0
YDL161W	YLR206W	ENT1	PSR2	non-RPG													0
YDL211C	YNL176C		SPH1	non-RPG													0
YDR028C	YBR050C	REG1	FRE2	non-RPG													0
YDR066C	YER139C	RTR2	SAM3	non-RPG													0
YDR146C	YLR131C	SWI5		non-RPG													0
YDR213W	YLR228C	UPC2	IDP3	non-RPG													0
YDR247W	YPL026C	VHS1	HEF3	non-RPG													0
YDR253C	YPL038W	MET32	LCB4	non-RPG													0
YDR263C	YOR033C	DIN7	YRR1	non-RPG													0
YDR264C	YOR034C	AKR1	GAC1	non-RPG													0
YDR368W	YOR120W	YPR1	DCI1	non-RPG													0
YDR379W	YOR127W	RGA2	CMP2	non-RPG													0
YDR441C	YML022W	APT2	ORC1	non-RPG													0
YDR490C	YOL100W	PKH1	HMG1	non-RPG													0
YEL022W	YJR031C	GEA2		non-RPG													0
YEL030W	YJR045C	ECM10		non-RPG													0
YER045C	YIL036W	ACA1	URA10	non-RPG													0
YER081W	YIL074C	SER3	BUL1	non-RPG													0
YER129W	YGL179C	SAK1	NGL2	non-RPG													0
YER132C	YGL197W	PMD1	PLB3	non-RPG													0
YFR015C	YLR258W	GSY1	GRE1	non-RPG													0
YGL071W	YPL202C	AFT1	CET1	non-RPG													0
YGL075C	YPL200W	MPS2		non-RPG													0
YGL096W	YPL177C	TOS8	SSO1	non-RPG													0
YGL166W	YPR008W	CUP2	GYP5	non-RPG													0
YGR004W	YLR324W	PEX31	VIK1	non-RPG													0
YGR023W	YLR332W	MTL1	CLN2	non-RPG													0
YGR142W	YPR158W	BTN2	DDR2	non-RPG													0
YGR192C	YJR009C	TDH3		non-RPG													0
YGR230W	YHR152W	BNS1	QDR1	non-RPG													0
YGR239C	YHR160C	PEX21	VPS21	non-RPG													0
YHL003C	YKL008C	LAG1	CAN1	non-RPG													0
YHL017W	YKL039W		WSC3	non-RPG													0
YHL035C	YLL048C	VMR1	MSB4	non-RPG													0
YHR096C	YDR345C	HXT5	SKM1	non-RPG													0
YHR139C	YNL160W	SPS100	PAP2	non-RPG													0
YIR018W	YOL028C	YAP5		non-RPG													0
YJL098W	YKR028W	SAP185	ERP2	non-RPG													0
YJL099W	YKR027W	CHS6	PDR15	non-RPG													0
YJL112W	YKR036C	MDV1		non-RPG													0
YJL133W	YKR052C	MRS3	ODC1	non-RPG													0
YJL134W	YKR053C	LCB3	ISU1	non-RPG													0
YJL138C	YKR059W	TIF2	MKK2	non-RPG													0
YJL159W	YKL163W	HSP150	SVS1	non-RPG													0
YJL160C	YKL164C		FRE4	non-RPG													0
YJR091C	YPR042C	JSN1	PEX27	non-RPG													0
YJR127C	YML081W	RSF2		non-RPG													0
YJR130C	YML082W	STR2	ICY1	non-RPG													0
YJR148W	YHR208W	BAT2	RIF2	non-RPG													0
YKL050C	YMR031C		PSK2	non-RPG													0
YKL062W	YMR037C	MSN4	MYO2	non-RPG													0
YKL068W	YMR047C	NUP100		non-RPG													0
YKL126W	YMR104C	YPK1	DOT6	non-RPG													0
YKR077W	YOR066W	MSA2	SSA4	non-RPG													0
YLR293C	YOR185C	GSP1		non-RPG													0

Gene.1	Gene.2	Name.1	Name.2	Gene class	Calb	Lklu	Lthe	Lwal	Egos	Klac	Zrou	Vpol	Ncas	Cgla	Sbay	Scer	preWGD Ancestor
YML125C	YML087C	PGA3	MRH1	non-RPG													0
YMR119W	YNL008C	ASI1	TEF1	non-RPG													0
YMR206W	YNR014W		SUR1	non-RPG													0
YNL095C	YOR092W		SYH1	non-RPG													0
YNL104C	YOR108W	LEU4	UBX6	non-RPG													0
YNL309W	YOL131W	STB1	GIN4	non-RPG													0
YNR034W-A	YCR075W-A		GNP1	non-RPG													0
YOR227W	YPL137C	HER1	GRX2	non-RPG													0
YOR237W	YPL145C	HES1	EMI2	non-RPG													0
YOR256C	YPL176C	TRE2	EUG1	non-RPG													0
YDR077W	YER150W	SED1	SEC12	non-RPG													0
YDR343C	YHR094C	HXT6	CPR8	non-RPG													0
YGR197C	YJR015W	SNG1		non-RPG													0
YIL013C	YOR011W	PDR11	GPM3	non-RPG													0
YKL093W	YMR081C	MBR1	ROG1	non-RPG													0
YFL059W	YNL333W	SNZ3	ENT2	non-RPG													0
YBL089W	YER119C	AVT5	TDA7	non-RPG													0
YCL050C	YDR530C	APA1	REG2	non-RPG													0
YDL022W	YOL059W	GPD1	RTR1	non-RPG													0
YDL214C	YNL183C	PRR2	ACE2	non-RPG													0
YDR272W	YOR040W	GLO2	ECM22	non-RPG													0
YDR524C-B	YCL048W-A		SKS1	non-RPG													0
YER037W	YGL224C	PHM8	MET31	non-RPG													0
YGR035C	YLR346C		EXO1	non-RPG													0
YGR221C	YHR149C	TOS2	AKR2	non-RPG													0
YGR279C	YMR305C	SCW4	GCY1	non-RPG													0
YHR117W	YNL121C	TOM71	RGA1	non-RPG													0
YIL105C	YNL047C	SLM1	APT1	non-RPG													0
YIL156W	YKR098C	UBP7	PKH2	non-RPG													0
YJL105W	YKR029C	SET4	GEA1	non-RPG													0
YJL165C	YKL168C	HAL5	SSC1	non-RPG													0
YKR075C	YOR062C		CST6	non-RPG													0
YLL012W	YLR020C	YEH1	SER33	non-RPG													0
YLR300W	YOR190W	EXG1	TOS3	non-RPG													0
YLR449W	YML074C	FPR4	MDS3	non-RPG													0
YML109W	YMR273C	ZDS2	GSY2	non-RPG													0
YMR177W	YPL224C	MMT1	AFT2	non-RPG													0
YMR186W	YPL240C	HSC82	CSM4	non-RPG													0
YDR252W	YPL037C	BTT1	CUP9	non-RPG													0
YIL085C	YNL029C	KTR7	HAA1	non-RPG													0
YAL023C	YOR321W	PMT2	PEX30	non-RPG													0
YAL038W	YOR347C	CDC19	MID2	non-RPG													0
YBL030C	YBR085W	PET9	CUR1	non-RPG													0
YBL056W	YER089C	PTC3	TDH2	non-RPG													0
YBR024W	YBR037C	SCO2	SPO12	non-RPG													0
YBR121C	YPR081C	GRS1	PEX18	non-RPG													0
YBR140C	YOL081W	IRA1	LAC1	non-RPG													0
YBR147W	YOL092W	RTC2	PTM1	non-RPG													0
YBR169C	YPL106C	SSE2	YBT1	non-RPG													0
YBR197C	YPL077C		HXT3	non-RPG													0
YBR210W	YGL054C	ERV15	YGP1	non-RPG													0
YBR241C	YGL104C		YAP7	non-RPG													0
YCL048W	YDR522C	SPS22	SAP190	non-RPG													0
YCL051W	YDR528W	LRE1	BCH2	non-RPG													0
YCR005C	YNR001C	CIT2	CAF4	non-RPG													0
YCR034W	YJL196C	FEN1	MRS4	non-RPG													0
YCR037C	YJL198W	PHO87	YSR3	non-RPG													0
YCR073C	YNR031C	SSK22	TIF1	non-RPG													0
YDR122W	YLR096W	KIN1	PIR3	non-RPG													0
YDR185C	YLR168C	UPS3	PIR1	non-RPG													0
YDR200C	YLR238W	VPS64	PUF2	non-RPG													0
YDR273W	YOR042W	DON1	TDA9	non-RPG													0

Gene.1	Gene.2	Name.1	Name.2	Gene class	Calb	Lklu	Lthe	Lwal	Egos	Klac	Zrou	Vpol	Ncas	Cgla	Sbay	Scer	preWGD Ancestor
YDR341C	YHR091C			non-RPG													0
YDR409W	YOR156C	SIZ1	BAT1	non-RPG													0
YDR451C	YML027W	YHP1	EIS1	non-RPG													0
YDR480W	YPL049C	DIG2	MSN2	non-RPG													0
YDR483W	YPL053C	KRE2	NUP116	non-RPG													0
YDR501W	YLR183C	PLM2	YPK2	non-RPG													0
YEL034W	YJR047C	HYP2	MSA1	non-RPG													0
YEL039C	YJR048W	CYC7	GSP2	non-RPG													0
YER064C	YIL056W		AIM33	non-RPG													0
YFL004W	YPL019C	VTC2	ASI3	non-RPG													0
YFR022W	YOR018W	ROG3		non-RPG													0
YFR040W	YGL229C	SAP155	ECM3	non-RPG													0
YGL084C	YPL189W	GUP1	LEU9	non-RPG													0
YGR008C	YLR327C	STF2		non-RPG													0
YGR092W	YPR111W	DBF2		non-RPG													0
YGR109C	YPR120C	CLB6	GIP3	non-RPG													0
YGR273C	YMR295C		KES1	non-RPG													0
YGR283C	YMR310C		TRE1	non-RPG													0
YHL034C	YLL046C	SBP1	SPI1	non-RPG													0
YIL095W	YNL020C	PRK1	HXT1	non-RPG													0
YIR033W	YKL020C	MGA2		non-RPG													0
YJL084C	YKR021W	ALY2	AUS1	non-RPG													0
YJR066W	YKL203C	TOR1	ISF1	non-RPG													0
YKL127W	YMR105C	PGM1	SNZ2	non-RPG													0
YKR089C	YOR081C	TGL4	AVT6	non-RPG													0
YKR091W	YOR083W	SRL3	APA2	non-RPG													0
YLR028C	YMR120C	ADE16	GPD2	non-RPG													0
YLR187W	YNL278W	SKG3	NPR1	non-RPG													0
YLR437C	YML058W	DIF1	GLO4	non-RPG													0
YML100W	YMR261C	TSL1		non-RPG													0
YMR243C	YOR316C	ZRC1	SDT1	non-RPG													0
YMR264W	YML101C	CUE1		non-RPG													0
YNL307C	YOL128C	MCK1	SKG6	non-RPG													0
YOL091W	YBR148W	SPO21	SCW10	non-RPG													0
YOR044W	YDR275W	IRC23	TOM70	non-RPG													0
YBL085W	YER114C	BOI1	SLM2	non-RPG													0
YBR042C	YDR018C	CST26	UBP11	non-RPG													0
YBR066C	YDR043C	NRG2	SET3	non-RPG													0
YBR216C	YGL060W	YBP1	KKQ8	non-RPG													0
YBR218C	YGL062W	PYC2		non-RPG													0
YDR069C	YER144C	DOA4	YEH2	non-RPG													0
YDR147W	YLR133W	EKI1	SPR1	non-RPG													0
YDR453C	YML028W	TSA2	FPR3	non-RPG													0
YDR475C	YOR019W	JIP4	ZDS1	non-RPG													0
YER019C-A	YER087C-B	SBH2	MMT2	non-RPG													0
YGL089C	YPL187W	MF(ALPHA)2	HSP82	non-RPG													0
YHL012W	YKL035W		EGD1	non-RPG													0
YJR054W	YML047C		KTR5	non-RPG													0
YKL072W	YMR053C	STB6	PMT3	non-RPG													0
YKR078W	YOR069W		PYK2	non-RPG													0
YLR270W	YOR173W	DCS1	AAC3	non-RPG													0
YML007W	YDR423C	YAP1	PTC2	non-RPG													0
YMR246W	YOR317W	FAA4	SCO1	non-RPG													0
YNL106C	YOR109W	INP52	GRS2	non-RPG													0
YOR204W	YPL119C	DED1	IRA2	non-RPG													0
YOR233W	YPL141C	KIN4		non-RPG													0
YDL088C	YMR153W	ASM4	SSE1	non-RPG													0
YDR304C	YHR057C	CPR5		non-RPG													0
YER059W	YIL050W	PCL6	ERV14	non-RPG													0
YHR003C	YKL027W		VPS73	non-RPG													0
YBR052C	YDR032C	RFS1	SPS2	non-RPG													0
YKL032C	YMR072W	IXR1	HLR1	non-RPG													0

Gene.1	Gene.2	Name.1	Name.2	Gene class	Calb	Lklu	Lthe	Lwal	Egos	Klac	Zrou	Vpol	Ncas	Cgla	Sbay	Scer	preWGD Ancestor
YKL201C	YJR061W	MNN4	CIT1	non-RPG													0
YNL195C	YDL223C		ELO1	non-RPG													0
YBR020W	YDR009W	GAL1	PHO90	non-RPG													0
YCR010C	YNR002C	ADY2	SSK2	non-RPG													0
YML116W	YMR279C	ATR1	KIN2	non-RPG													0
YAL028W	YOR324C	FRT2	UPS2	non-RPG													0
YBR242W	YGL101W		FAR10	non-RPG													0
YCL073C	YKR106W	GEX1	CUE5	non-RPG													0
YCR091W	YNR047W	KIN82	MSR1	non-RPG													0
YDR385W	YOR133W	EFT2	NFI1	non-RPG													0
YGR188C	YJL013C	BUB1	YOX1	non-RPG													0
YGR243W	YHR162W	FMP43	DIG1	non-RPG													0
YHR131C	YNL144C		KTR6	non-RPG													0
YJL137C	YKR058W	GLG2	TOS4	non-RPG													0
YAL053W	YOR365C	FLC2	ANB1	non-RPG													0
YGR180C	YJL026W	RNR4	CYC1	non-RPG													0
YNL087W	YOR086C	TCB2	VHR1	non-RPG													0
YDL182W	YDL131W	LYS20	VTC3	non-RPG													0
YHR133C	YNL156C	NSG1	ROD1	non-RPG													0
YIL114C	YNL055C	POR2	SAP4	non-RPG													0
YGR248W	YHR163W	SOL4	GUP2	non-RPG													0
YGR136W	YPR154W	LSB1	TMA10	non-RPG													0
YGL158W	YLR248W	RCK1	DBF20	non-RPG													0
YOR229W	YPL139C	WTM2	CLB5	non-RPG													0
YGL063W	YPL212C	PUS2		non-RPG													0
YBR005W	YDR003W	RCR1		non-RPG													0
YBR182C	YPL089C	SMP1	RNP1	non-RPG													0
YGR256W	YHR183W	GND2	ARK1	non-RPG													0
YIL151C	YKR096W		SPT23	non-RPG													0
YMR233W	YOR295W	TRI1	ALY1	non-RPG													0
YBR238C	YGL107C		TOR2	non-RPG													0
YGR032W	YLR342W	GSC2	PGM2	non-RPG													0
YGL119W	YBR230W-A	COQ8	TGL5	non-RPG													0
YIL131C	YNL068C	FKH1	WHI5	non-RPG													0
YKR084C	YOR076C	HBS1	ADE17	non-RPG													0
YNL098C	YOR101W	RAS2	CAF120	non-RPG													0
YMR237W	YOR299W	BCH1	SML1	non-RPG													0
YNL108C	YOR110W		TPS3	non-RPG													0
YLR277C	YOR179C	YSH1	COT1	non-RPG													0
YER031C	YGL210W	YPT31	CUE4	non-RPG													0
YIL138C	YNL079C	TPM2	YGK3	non-RPG													0
YMR207C	YNR016C	HFA1	YSW1	non-RPG													0
YMR182C	YPL230W	RGM1	BSC2	non-RPG													0
YCL011C	YNL004W	GBP2	BOI2	non-RPG													0
YBR089C-A	YPR052C	NHP6B		non-RPG													0
YDL079C	YMR139W	MRK1	NRG1	non-RPG													0
YKL091C	YMR079W		YBP2	non-RPG													0
YHR047C	YKL157W	AAP1	PYC1	non-RPG													0
YBL091C-A	YER120W	SCS22	UBP5	non-RPG													0
YDR099W	YER177W	BMH2	CKI1	non-RPG													0
YDR458C	YML034W	HEH2	TSA1	non-RPG													0
YBR078W	YDR055W	ECM33		non-RPG													0
YDL012C	YBR016W		SBH1	non-RPG													0
YDL137W	YDL192W	ARF2	MF(ALPHA)1	non-RPG													0
YAL030W	YOR327C	SNC1	UGP1	non-RPG													0
YDR348C	YHR097C		PRM6	non-RPG													0
YML056C	YLR432W	IMD4	STB2	non-RPG													0
YNL052W	YIL111W	COX5A	VPS5	non-RPG													0
Scer_snR17a	Scer_snR17b	SNR17A	DCS2	non-RPG													0
YHR123W	YNL130C	EPT1	CAD1	non-RPG													0
YBR082C	YDR059C	UBC4	FAA1	non-RPG													0
YBL059W	YER093C-A		INP53	non-RPG													0

Gene.1	Gene.2	Name.1	Name.2	Gene class	Calb	Lklu	Lthe	Lwal	Egos	Klac	Zrou	Vpol	Ncas	Cgla	Sbay	Scer	preWGD Ancestor
YML124C	YML085C	TUB3	DBP1	non-RPG													0
YFR024C-A	YHR016C	LSB3	FRK1	non-RPG													0
YIL123W	YNL066W	SIM1	NUP53	non-RPG													0
YBR031W	YDR012W	RPL4A	CPR2	non-RPG													0
YDR418W	YEL054C	RPL12B	PCL7	non-RPG													0
YGL135W	YPL220W	RPL1B		non-RPG													0
YLR441C	YML063W	RPS1A	PST2	non-RPG													0
YLR029C	YMR121C	RPL15A	ABF2	non-RPG													0
YGR085C	YPR102C	RPL11B		non-RPG													0
YDL184C	YDL133C-A	RPL41A	HBT1	non-RPG													0
YHL033C	YLL045C	RPL8A	GAL3	non-RPG													0
YLR264W	YOR167C	RPS28B	ATO2	non-RPG													0
YHL001W	YKL006W	RPL14B		non-RPG													0
YJL177W	YKL180W	RPL17B	FRT1	non-RPG													0
YDR471W	YHR010W	RPL27B		non-RPG													0
YER131W	YGL189C	RPS26B	GEX2	non-RPG													0
YIL148W	YKR094C	RPL40A	FPK1	non-RPG													0
YJR094W-A	YPR043W	RPL43B	EFT1	non-RPG													0
YMR230W	YOR293W	RPS10B	MAD3	non-RPG													0
YDL082W	YMR142C	RPL13A		non-RPG													0
YER074W	YIL069C	RPS24A		non-RPG													0
YFR031C-A	YIL018W	RPL2A	GLG1	non-RPG													0
YGL031C	YGR148C	RPL24A		non-RPG													0
YGR118W	YPR132W	RPS23A	RNR2	non-RPG													0
YHR021C	YKL156W	RPS27B	TCB1	non-RPG													0
YIL133C	YNL069C	RPL16A	LYS21	non-RPG													0
YNL096C	YOR096W	RPS7B	NSG2	non-RPG													0
YHR203C	YJR145C	RPS4B	POR1	non-RPG													0
YBL027W	YBR084C-A	RPL19B	SOL3	non-RPG													0
YBL072C	YER102W	RPS8A	PIN3	non-RPG													0
YBL087C	YER117W	RPL23A	RCK2	non-RPG													0
YBR048W	YDR025W	RPS11B	UME1	non-RPG													0
YBR181C	YPL090C	RPS6B	RCR2	non-RPG													0
YBR189W	YPL081W	RPS9B	RLM1	non-RPG													0
YBR191W	YPL079W	RPL21A	GND1	non-RPG													0
YCR031C	YJL191W	RPS14A		non-RPG													0
YDL061C	YLR388W	RPS29B	UAF30	non-RPG													0
YDL075W	YLR406C	RPL31A	RMD9	non-RPG													0
YDL083C	YMR143W	RPS16B	FKH2	non-RPG													0
YDL191W	YDL136W	RPL35A	RAS1	non-RPG													0
YDR447C	YML024W	RPS17B	BUD7	non-RPG													0
YDR450W	YML026C	RPS18A	TFC7	non-RPG													0
YDR500C	YLR185W	RPL37B	SYC1	non-RPG													0
YER056C-A	YIL052C	RPL34A	TPM1	non-RPG													0
YFL034C-A	YLR061W	RPL22B	ACC1	non-RPG													0
YGR027C	YLR333C	RPS25A	USV1	non-RPG													0
YGR034W	YLR344W	RPL26B	BMH1	non-RPG													0
YGR214W	YLR048W	RPS0A	SNR17B	non-RPG													0
YHR141C	YNL162W	RPL42B	RPL4B	RPG													0
YJL136C	YKR057W	RPS21B	RPL12A	RPG													0
YLR287C-A	YOR182C	RPS30A	RPL1A	RPG													0
YLR448W	YML073C	RPL6B	RPS1B	RPG													0
YMR194W	YPL249C-A	RPL36A	RPL15B	RPG													0
YMR242C	YOR312C	RPL20A	RPL11A	RPG													0
YNL301C	YOL120C	RPL18B	RPL41B	RPG													0
YNL302C	YOL121C	RPS19B	RPL8B	RPG													0
YOR234C	YPL143W	RPL33B	RPS28A	RPG													0
YGL076C	YPL198W	RPL7A	RPL7B	RPG													0

Code	WGD	Intron evidence	Intron number	Gene number
	pre-WGD	none	0	1
	pre-WGD	1 annotation	1	1
	pre-WGD	1 prediction	1	1
	pre-WGD	1 annotation	multiple per gene	1
	post-WGD	none	0	2
	post-WGD	prediction	1	2
	post-WGD	annotation	1	2
	post-WGD	1 prediction and 1 annotation	2	2
	post-WGD	2 predictions	2	2
	post-WGD	2 annotation	2	2
	post-WGD	annotation	multiple per gene	2
	post-WGD	none	0	1
	post-WGD	1 predictions	1	1
	post-WGD	1 annotation	1	1
	No ortholog	NA	NA	NA
