# Supplementary material for: Diverse Forms of RPS9 Splicing Are Part of an Evolving Autoregulatory Circuit
Source: PLoS Genet. 2012 Mar 29;8(3):e1002620. doi: 10.1371/journal.pgen.1002620 (PMC3315480; doi:10.1371/journal.pgen.1002620)
Supplement: Table S2 — Primers. (PDF) [file pgen.1002620.s006.pdf]

| Organism | Template | Amplicon | Forward Primer | Reverse Primer | Technique | Figure |
|---|---|---|---|---|---|---|
| Sc | pJPS1232 | RPL16 Round 1 PCR | aacaaatcgagaaaatgtctgttgaaccagttgttgtcattgatgGTAAGGGTCATTTAGTAGGTCGTTT | gatgctcattcaaaaaaatggatttgttagtattatcctttaaatTCCTTACCATTAAGTTGATC | intron deletion | 2A |
| Sc | pJPS1232 | RPL16 Round 2 PCR | gtaagggtcatttagtaggtcgtttagcttccgttgttgctaagcGTATTACCCTGTTATCCCTA | gatgctcattcaaaaaaatggatttgttagtattatcctttaaatTCCTTACCATTAAGTTGATC | intron deletion | 2A |
| Sc | pJPS1232 | RPL23A Round 1 PCR | ataatgtccggtaacggtgctcaaggtactaagtttagaatctcaTTAGGTCTACCAGTCGGTGCCATCA | gagaataattttgttagtaaaagcatagggagaaagttagcagatTCCTTACCATTAAGTTGATC | intron deletion | 2A |
| Sc | pJPS1232 | RPL23A Round 2 PCR | ttaggtctaccagtcggtgccatcatgaactgtgctgacaacagtGTATTACCCTGTTATCCCTA | gagaataattttgttagtaaaagcatagggagaaagttagcagatTCCTTACCATTAAGTTGATC | intron deletion | 2A |
| Sc | pJPS1232 | RPL24A Round 1 PCR | tgctattgccaagttaagcaagacaccaagacaataacttgagagTGATAAAATGAAGGTTGAAATCGAT | gaaaaaaaaaatgttagtaagtctatgcttttcataggtagccatTCCTTACCATTAAGTTGATC | intron deletion | 2A |
| Sc | pJPS1232 | RPL24A Round 2 PCR | tgataaaatgaaggttgaaatcgattctttttcaggtgccaaaatGTATTACCCTGTTATCCCTA | gaaaaaaaaaatgttagtaagtctatgcttttcataggtagccatTCCTTACCATTAAGTTGATC | intron deletion | 2A |
| Sc | pJPS1232 | RPL2A Round 1 PCR | ccatttctgagcaaactaagaaaccattagatcaataagcaatggGTAGAGTTATTCGTAACCAAAGAAA | tctgttagtatacacgatacattttcaacaaaaggaataaaaagaTCCTTACCATTAAGTTGATC | intron deletion | 2A |
| Sc | pJPS1232 | RPL2A Round 2 PCR | gtagagttattcgtaaccaaagaaagggtgctggttctatctttaGTATTACCCTGTTATCCCTA | tctgttagtatacacgatacattttcaacaaaaggaataaaaagaTCCTTACCATTAAGTTGATC | intron deletion | 2A |
| Sc | pJPS1232 | RPL2B Round 1 PCR | tttgtcataaactcaccaagaaaccacaaagttattgaacaatggGTAGAGTTATTCGTAACCAAAGAAA | tgttagtaaatatttctcgaaagtttttttcattatcaaaatatggTCCTTACCATTAAGTTGATC | intron deletion | 2A |
| Sc | pJPS1232 | RPL2B Round 2 PCR | gtagagttattcgtaaccaaagaaagggtgctggttctatcttcaGTATTACCCTGTTATCCCTA | tgttagtaaatatttctcgaaagtttttttcattatcaaaatatggTCCTTACCATTAAGTTGATC | intron deletion | 2A |
| Sc | pJPS1232 | RPL13A Round 1 PCR | tttgagaaaagcatagtacagcacagcaggaatcgtacacaatggCCATTTCCAAGAATTTACCAATCTT | aaaaattgttagtatacagaaaagtggacgtttcaattagacaccTCCTTACCATTAAGTTGATC | intron deletion | 2A |
| Sc | pJPS1232 | RPL13A Round 2 PCR | ccatttccaagaatttaccaatcttaaagaatcatttcagaaagcGTATTACCCTGTTATCCCTA | aaaaattgttagtatacagaaaagtggacgtttcaattagacaccTCCTTACCATTAAGTTGATC | intron deletion | 2A |
| Sc | pJPS1232 | RPL34A Round 1 PCR | aaccaaagatggcccaacgtgttactttcagaagaagaaatccatACAACACCCGTTCTAACAAAATCAA | actttctcatttgaaaaatatgttagtacgaaatgtttcaataggTCCTTACCATTAAGTTGATC | intron deletion | 2A |
| Sc | pJPS1232 | RPL34A Round 2 PCR | acaacacccgttctaacaaaatcaaggttgttaagaccccaggtgGTATTACCCTGTTATCCCTA | actttctcatttgaaaaatatgttagtacgaaatgtttcaataggTCCTTACCATTAAGTTGATC | intron deletion | 2A |
| Sc | pJPS1232 | RPL35A Round 1 PCR | ttattgaccgtagaataggtacagtgagacagtatattcgaaatgGCCGGTGTTAAAGCTTACGAACTAA | ccaaatttttattaacgttagtattttattagcacttataatgaaTCCTTACCATTAAGTTGATC | intron deletion | 2A |
| Sc | pJPS1232 | RPL35A Round 2 PCR | gccggtgttaaagcttacgaactaagaaccaaatccaaggaacaaGTATTACCCTGTTATCCCTA | ccaaatttttattaacgttagtattttattagcacttataatgaaTCCTTACCATTAAGTTGATC | intron deletion | 2A |
| Sc | pJPS1232 | RPL40B Round 1 PCR | cccgttcagcaagaggtaaagccaccaaaggttcaaaatgcaaatCTTCGTTAAGACTTTGACTGGTAAG | agtaagcacagacgtgcgtgcgtacatcataaagaaagaaacttcTCCTTACCATTAAGTTGATC | intron deletion | 2A |
| Sc | pJPS1232 | RPL40B Round 2 PCR | cttcgttaagactttgactggtaagaccatcactttggaagttgaGTATTACCCTGTTATCCCTA | agtaagcacagacgtgcgtgcgtacatcataaagaaagaaacttcTCCTTACCATTAAGTTGATC | intron deletion | 2A |
| Sc | pJPS1232 | RPSS14A Round 1 PCR | ttaaacccaaggagagtaagaaaccacaagaacccgccatgtctaACGTTGTTCAAGCTCGTGACAATTC | atatcaaacgtaatgttgttagtatccaaaagttttttttttaaTCCTTACCATTAAGTTGATC | intron deletion | 2A |
| Sc | pJPS1232 | RPSS14A Round 2 PCR | acgttgttcaagctcgtgacaattcccaagtttttggtgttgctaGTATTACCCTGTTATCCCTA | atatcaaacgtaatgttgttagtatccaaaagttttttttttaaTCCTTACCATTAAGTTGATC | intron deletion | 2A |
| Sc | pJPS1232 | RPS14B Round 1 PCR | agactgaaatcaacaactcccaataacaattaagaatggctaacgACCTTGTTCAAGCTCGCGATAACTC | aacagaacttctgctttttcgattattaaaaaataaacgaatgagTCCTTACCATTAAGTTGATC | intron deletion | 2A |
| Sc | pJPS1232 | RPS14B Round 2 PCR | accttgttcaagctcgcgataactctcaagtctttggtgttgccaGTATTACCCTGTTATCCCTA | aacagaacttctgctttttcgattattaaaaaataaacgaatgagTCCTTACCATTAAGTTGATC | intron deletion | 2A |
| Sc | pJPS1232 | RPS21B Round 1 PCR | aaagaatatcaataagccaaaatggaaaacgataagggtcaattaGTTGAATTATATGTTCCAAGAAAGT | acatttgcgcttatattcttgtaaaattattctgcacatgaatgaTCCTTACCATTAAGTTGATC | intron deletion | 2A |
| Sc | pJPS1232 | RPS21B Round 2 PCR | gttgaattatatgttccaagaaagtgttctgctaccaacagaatcGTATTACCCTGTTATCCCTA | acatttgcgcttatattcttgtaaaattattctgcacatgaatgaTCCTTACCATTAAGTTGATC | intron deletion | 2A |
| Sc | pJPS1232 | RPS23A Round 1 PCR | aggtttgaactctgctagaaagctacgtgtccacagaagaaacaaCCGTTGGGCCGAAAACAACTACAAG | aaatgattccgttgtaaaaaacggcctcctgtgactttctgagttTCCTTACCATTAAGTTGATC | intron deletion | 2A |
| Sc | pJPS1232 | RPS23A Round 2 PCR | ccgttgggccgaaaacaactacaagaagagattgttgggtactgcGTATTACCCTGTTATCCCTA | aaatgattccgttgtaaaaaacggcctcctgtgactttctgagttTCCTTACCATTAAGTTGATC | intron deletion | 2A |
| Sc | pJPS1232 | RPS8A Round 1 PCR | ttttggtactcacaagaagacgttataaacacaccaggacaaaaaTTACAAAATGGGTATTTCTCGTGAT | taaaataaagaacgtacaaaagttgcggaatttttttcttatcttTCCTTACCATTAAGTTGATC | intron deletion | 2A |
| Sc | pJPS1232 | RPS8A Round 2 PCR | ttacaaaatgggtatttctcgtgattctcgtcacaaaagatccgcGTATTACCCTGTTATCCCTA | taaaataaagaacgtacaaaagttgcggaatttttttcttatcttTCCTTACCATTAAGTTGATC | intron deletion | 2A |
| Sc | pJPS1232 | RPS9A Round 1 PCR | aaaactaatacagcaacagaaatacaaaagtatacaacatgccaaGAGCCCCAAGAACATATTCCAAGAC | gtgagcgctctctttttcaaacgaatgcgaggatcaagatcactaTCCTTACCATTAAGTTGATC | intron deletion | 2A |
| Sc | pJPS1232 | RPS9A Round 2 PCR | gagccccaagaacatattccaagacttactctaccccaaagagacGTATTACCCTGTTATCCCTA | gtgagcgctctctttttcaaacgaatgcgaggatcaagatcactaTCCTTACCATTAAGTTGATC | intron deletion | 2A |
| Sc | pJPS1232 | RPS9B Round 1 PCR | aagaaagaaagcaacaagacaactaagactaagcaacaatgccaaGAGCTCCAAGAACTTACTCTAAGAC | ttagtcaaaagttttcccggaacaaaagtctgcaagggcccaagtTCCTTACCATTAAGTTGATC | intron deletion | 2A |
| Sc | pJPS1232 | RPS9B Round 2 PCR | gagctccaagaacttactctaagacttactctacaccaaagagacGTATTACCCTGTTATCCCTA | ttagtcaaaagttttcccggaacaaaagtctgcaagggcccaagtTCCTTACCATTAAGTTGATC | intron deletion | 2A |
| Sc | cDNA | Total RPS9A | ccaacatctccattcggtgg | aaaatcagcacaattattcttca | qPCR | 2D |
| Sc | cDNA | Total RPS9B | gctagaagaaacgctgctaga | tgaaaaatgatccgtgatttga | qPCR | 2D |
| Sc | cDNA | SCR1 | aggaatccgtctctctgtct | TGGATAAAACTCCCCTAACA | qPCR | 2D |
| Dm | cDNA | UPF1 1 | GGGCGGGTgcctctcgcaaagtttgttc | GGGCGGGTgttagatcgcacgcaagaca | RNAi | 3A |
| Dm | cDNA | UPF1 2 | GGGCGGGTtggtccctacggaaactcac | GGGCGGGTccgagcttccttgtgttagc | RNAi | 3A |
| Dm | cDNA | HNRNPLL | GGGCGGGTgatcactcggccaggaaata | GGGCGGGTaccagagggatttcctccat | RNAi | 3A |
| Dm | cDNA | RPLP0 | agaagaaggaggaggccaag | ccgaaacccatatcatcgtc | qPCR | 3B |
| Dm | cDNA | RPS9 PTC+ | tgactccgttcacaccaatc | tcaagtctgcgccatgtatt | qPCR | 3B |
| Dm | cDNA | Total exogenous RPS9 | gtcgcgtcaagaggaagaac | cctgctagcttacgtcacca | qPCR | 3B |
| Dm | cDNA | Total endogenous RPS9 | gtcgcgtcaagaggaagaac | tcaagtctgcgccatgtatt | qPCR | 3B |
| Dm | cDNA | GAPDH1 | cttcttcagcgacacccatt | accgaactcgttgtcgtacc | qPCR | 3B |
| Dm | cDNA | RPS9 cDNA ORF | CACCatggtgaacggccgcatac | ttagtcctcctcctcttcagc | subcloning | 3B |
